# Protocol using similarity score and improved shrink-wrap algorithm for better convergence of phase-retrieval calculation in X-ray diffraction imaging

**DOI:** 10.1107/S1600577523009864

**Published:** 2024-01-01

**Authors:** Syouyo Yoshida, Kosei Harada, So Uezu, Yuki Takayama, Masayoshi Nakasako

**Affiliations:** a Department of Physics, Faculty of Science and Technology, Keio University, 3-14-1 Hiyoshi, Kohoku-ku, Yokohama 223-8522, Japan; b RIKEN Spring-8 Center, 1-1-1 Kouto, Sayo-cho, Sayogun, Hyogo, Japan; c International Center for Synchrotron Radiation Innovation Smart, Tohoku University, Katahira 2-1-1, Aoba-ku, Sendai 980-8577, Japan; RIKEN SPring-8 Center, Japan

**Keywords:** X-ray diffraction imaging, phase-retrieval calculation, structure analysis of non-crystalline particles, X-ray free-electron laser

## Abstract

In X-ray diffraction imaging (XDI), electron density maps of a targeted particle are reconstructed computationally from the diffraction pattern alone using phase-retrieval (PR) algorithms. However, the PR calculations sometimes fail to yield realistic electron density maps that approximate the structure of the particle. This occurs due to the absence of structure amplitudes at and near the zero-scattering angle and the presence of Poisson noise in weak diffraction patterns. Consequently, the PR calculation becomes a bottleneck for XDI structure analyses. Here, a protocol to efficiently yield realistic maps is proposed. The protocol is based on the empirical observation that realistic maps tend to yield low similarity scores, as suggested in our prior study [Sekiguchi *et al.* (2017), *J. Synchrotron Rad.* **24**, 1024–1038]. Among independently and concurrently executed PR calculations, the protocol modifies all maps using the electron-density maps exhibiting low similarity scores. This approach, along with a new protocol for estimating particle shape, improved the probability of obtaining realistic maps for diffraction patterns from various aggregates of colloidal gold particles, as compared with PR calculations performed without the protocol. Consequently, the protocol has the potential to reduce computational costs in PR calculations and enable efficient XDI structure analysis of non-crystalline particles using synchrotron X-rays and X-ray free-electron laser pulses.

## Abstract 2

A protocol to steer phase-retrieval calculations to realistic density maps is proposed.

## 1. Introduction

X-ray diffraction imaging (XDI) is a technique used to visualize the structures of non-crystalline particles in the fields of biology and material sciences, with dimensions ranging from sub-micrometre to micrometre (Miao *et al.*, 1999 ▸, 2015 ▸; Nakasako, 2018 ▸). In an XDI experiment, a spatially isolated non-crystalline particle is irradiated by X-rays with almost complete spatial coherence, and the diffraction pattern is recorded in the far-field under oversampling (OS) conditions (Miao *et al.*, 2003*a* ▸). The electron density map of the particle, projected along the direction of the incident X-rays, is, in principle, computationally retrieved from the structure amplitude alone using iterative phase-retrieval (PR) algorithms (Fienup, 1982 ▸). Therefore, XDI has been applied to visualize the shapes and internal structures of non-crystalline particles that cannot be crystallized in the fields of material sciences and biology. This is accomplished using synchrotron radiation X-rays (Williams *et al.*, 2003 ▸; Shapiro *et al.*, 2005 ▸; Miao *et al.*, 2006 ▸; Nishino *et al.*, 2009 ▸; Jiang *et al.*, 2010 ▸; Takayama & Nakasako, 2012 ▸; Nam *et al.*, 2013 ▸; Takayama *et al.*, 2018 ▸; Kobayashi *et al.*, 2018*a* ▸) and X-ray free-electron laser (XFEL) pulses (Seibert *et al.*, 2011 ▸; Loh *et al.*, 2012 ▸; Nakasako *et al.*, 2013 ▸; Takahashi *et al.*, 2013 ▸; Gallagher-Jones *et al.*, 2014 ▸; Hantke *et al.*, 2014 ▸; Xu *et al.*, 2014 ▸; Kimura *et al.*, 2014 ▸; Oroguchi *et al.*, 2015 ▸; Takayama *et al.*, 2015*a* ▸; van der Schot *et al.*, 2015 ▸; Ekeberg *et al.*, 2015 ▸; Kobayashi *et al.*, 2016*a* ▸; Kameda *et al.*, 2017 ▸; Oroguchi *et al.*, 2018 ▸; Nakasako, 2018 ▸; Nakasako *et al.*, 2020 ▸; Ayyer *et al.*, 2021 ▸; Cho *et al.*, 2021 ▸; Kobayashi *et al.*, 2021 ▸; Uezu *et al.*, 2023 ▸).

In XDI experiments, diffraction patterns at and around the zero-scattering angle are hidden by the beamstop and are missed in the strong intensity regions causing the saturation of area detectors (Martin *et al.*, 2012 ▸; Kobayashi *et al.*, 2014 ▸). In addition, diffraction patterns with weak intensities are susceptible to Poisson noise in X-ray detection, particularly at high diffraction angles. These factors reduce the efficiency of PR calculations in obtaining realistic projection electron density maps, which approximate the size, shape and internal structure of the specimen particle (Huang *et al.*, 2010 ▸; Kobayashi *et al.*, 2014 ▸; Sekiguchi *et al.*, 2016 ▸, 2017 ▸). As a result, despite the routine collection of diffraction patterns from non-crystalline particles at synchrotron and XFEL facilities (Martin *et al.*, 2012 ▸; Nakasako *et al.*, 2013 ▸; Kobayashi *et al.*, 2016*a* ▸, 2018*a* ▸; Takayama *et al.*, 2018 ▸; Nakasako *et al.*, 2020 ▸), the PR calculation remains a bottleneck in efficiently performing XDI structure analyses of non-crystalline particles.

To obtain realistic maps in PR calculations, various protocols have been proposed to modify the real-space constraint within the PR algorithm (Chen *et al.*, 2007 ▸; Marchesini *et al.*, 2003 ▸; Rodriguez *et al.*, 2013 ▸), as well as a protocol aimed at enhancing diffraction intensity (Takayama *et al.*, 2015*b* ▸). Additionally, a metric has been proposed for extracting realistic maps (Favre-Nicolin *et al.*, 2020 ▸). In our previous study, we introduced the application of multivariate analysis to phase-retrieved maps (Sekiguchi *et al.*, 2016 ▸). This analysis involves classifying maps obtained from 1000 independently conducted PR calculations within a space defined by a few key variables that predominantly capture the variations among the retrieved maps. Realistic maps are then identified from the classified maps through manual inspection.

To computationally extract realistic maps, we proposed the use of a similarity score (Miao *et al.*, 2003*b* ▸; Sekiguchi *et al.*, 2017 ▸), defined as

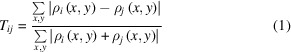

where ρ_*i*_(*x*, *y*) is the electron density distribution in the *i*th map. Based on empirical observations, when the similarity scores are below 0.2 it is likely that the pair of maps closely approximate the size, shape and internal structure of the specimen particle. As the similarity score and threshold are correlated with the phase differences between the structure factors of the two maps (Takayama & Nakasako, 2024 ▸), the use of (1 − *T*_*ij*_) should be avoided. While the similarity score can assist in automatically extracting realistic maps from a large number of PR maps, reducing the number of trial calculations remains challenging.

**Figure 1:**
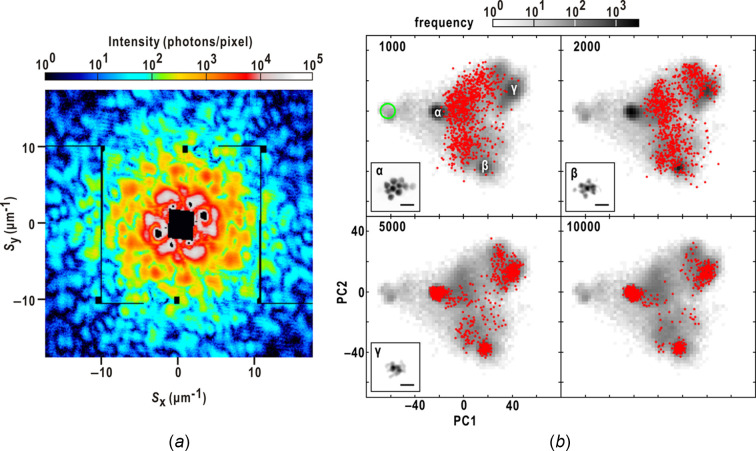
(*a*) Single-shot diffraction pattern from an aggregate of ten colloidal gold particles recorded using an XFEL pulse. The pattern displayed good centro-symmetry. (*b*) Progress of 840 PR calculations with the ordinary protocol (details are given in Section 3.5) for the diffraction pattern in panel (*a*). The position of each PR map (red dot) is plotted on the plane spanned by the first and second PC vectors (PC1 and PC2) obtained using PCA for the final 840 maps after the 10000 PR calculations (details are given in Section 3.8). The gray-scaled background map commonly shown in each panel displays how frequently PR maps visited each position on the plane during the 840 PR calculations. The scale of the frequency is presented at the top of the panel, and the number of PR cycles is labeled in the upper left of each panel. The green circle indicates the area of the 840 random maps for initiating independently three sets of 280 PR calculations. The PR maps in the three regions designated α, β and γ, where PR maps frequently resided, are depicted with a scale bar of 500 nm. Realistic maps from 52.6% of all the trial PR calculations appeared in region α only and yielded similarity scores smaller than 0.2. The diffraction pattern in our previous work (Sekiguchi *et al.*, 2017 ▸) was used with approval from the International Union of Crystallography.

To understand how maps change during the progress of PR calculations, we visualized the trajectory of maps on a plane defined by two principal component (PC) vectors obtained from the principal component analysis (PCA) of all PR maps (Fig. 1 ▸). The two vectors representatively characterized all PR maps. Initially, the maps were sparsely distributed, but gradually converged towards three distinct regions, with only one of them containing realistic maps. Interestingly, even in the early stages of PR calculations, several maps from different trials reached the region of realistic maps. These maps could potentially be utilized to modify all PR maps and guide subsequent rounds of PR. Our observations indicated that realistic maps tended to yield the smallest similarity scores among the PR maps, making it possible to automatically extract realistic maps using the similarity score.

Inspired by the visualized trajectory of PR calculations (Fig. 1 ▸), we proposed a protocol to steer PR calculations by iteratively modifying maps after specific cycles in parallel PR calculations. To evaluate the effectiveness and efficiency of the protocol, we applied the protocol to diffraction patterns obtained from aggregates of colloidal gold particles. These particles had known sizes and shapes, making them suitable for judging the reality of PR maps. The assessment demonstrated the effectiveness of the protocol to improve the probability to obtain realistic maps. This study has the potential to contribute not only to XDI but also to other fields that require PR calculations.

## 2. Protocol to steer PR calculation

**Figure 2:**
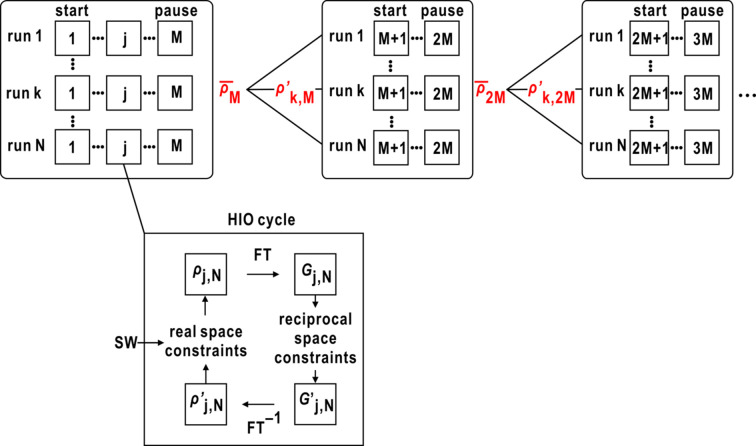
Schematic on the steered protocol. *N* independent PR calculations (runs) are parallelly conducted. At the lower left, the HIO (details are given in Section 3.5) calculation in the *j*th cycle is shown. The *j*th cycle of run *N* starts from the density map ρ_*j*,*N*_. The Fourier transform (FT) of ρ_*j*,*N*_, *G*_*j*,*N*_, is modified to 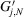 by the reciprocal space constraint (Kodama & Nakasako, 2011 ▸). The output map of the *j*th cycle 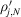 , which is calculated by the inverse Fourier transform (FT^−1^) of 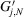 , is modified to ρ_*j*+1,*N*_ by the real-space constraint to start the next *j*+1 cycle. After *M* HIO cycles, the map for the next *M* + 1 cycle of the *k*th run, 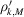 was generated using equations (2) and (3).

The protocol presented in this section is based on the assumption that pairs of maps with similarity scores smaller than 0.2 are likely to approximate the size, shape and internal structures of the specimen particle. To guide the PR calculations computationally, we execute *N* parallel PR calculation trials. After completing the first *M* cycles, all PR calculations are temporarily paused (Fig. 2 ▸). Subsequently, similarity scores are calculated for all pairs of *N* maps after aligning their centers of gravity. Among all map pairs, one of the pair displaying the smallest similarity score is tentatively selected as a reference map. Next, similarity scores for *N* − 1 maps against the reference are recalculated after optimally aligning the *N* − 1 maps to the reference map by applying translation and correcting for π-rotation caused by the center of symmetry in the diffraction pattern.

Among various modification methods tested, the protocol employing the following two equations has currently yielded the most favorable outcomes. From a set of maps with similarity scores smaller than 0.2, a map [ 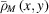 ] is generated for modifying all maps in the subsequent cycle using the following equation,

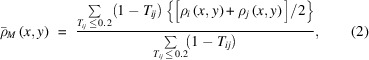

where ρ_*i*_(*x*, *y*) is *i*th map and yields the similarity score, *T*_*ij*_, smaller than 0.2 against the *j*th map, ρ_*j*_(*x*, *y*). In equation (2), it is important to note that even pairs of maps displaying electron densities different from the actual particle can still yield small similarity scores. Consequently, these maps can significantly contribute to the generation of 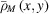 . Although it is possible for a pair of non-realistic maps to occasionally have the smallest similarity score among all PR maps in the early stages of the PR calculation, the sum for all the pairs of maps displaying similarity scores below 0.2 can be advantageous in reducing the influence of non-realistic maps on the modification process.

Using 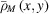 , the map from the *k*th trial calculation at the end of the *M*th cycle [ρ_*k*,*M*_(*x*, *y*)] is modified as

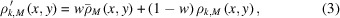

where *w* is a weight to control the contribution of 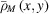 in the modification. In this study, after testing the weighting schemes, *w* started from 0.05 and increased by 0.05 at every modification stage until it reached 0.50. The cyclic increase of *w* serves as an effective strategy to mitigate the influence of non-realistic PR maps with low similarity scores. When the majority of the selected PR maps based on similarity scores are deemed realistic, equations (2) and (3) can modify all maps by leveraging the characteristics of the selected maps. This iterative modification process is applied to PR maps after several hundred cycles of PR calculations (Fig. 2 ▸). For applying the protocol in the initial stage of PR calculations, PR maps are better to have various density distributions to sample both the realistic and non-realistic PR maps. Therefore, we developed a protocol for mildly modifying the particle shape – details are given in Section 3.6.

Based on our experiences with typical PR calculation trials using diffraction patterns from aggregates of colloidal gold particles, it often requires more than 250 independent PR calculation trials, starting from different initial maps, to identify maps with similarity scores below 0.2. In cases where PR calculations continue to produce maps with similarity scores greater than 0.2 even after multiple rounds of modifications, the termination of the PR calculations may be more efficient in reducing computational costs.

## 3. Experimental procedure and phase-retrieval calculation

### 3.1. Specimen disk for XFEL-XDI experiments

In the XFEL-XDI experiment using the KOTOBUKI-1 diffraction apparatus (Nakasako *et al.*, 2013 ▸), a custom-made silicon disk of diameter 10 mm was used. The disk had a single 1 mm × 1 mm window covered by a 100 nm-thick Si_3_N_4_ membrane (Norcada, Canada). In experiments using the TAKASAGO-6 diffraction apparatus (Kobayashi *et al.*, 2016*a* ▸), we used a custom-made 8 mm × 10 mm silicon frame with nine 1 mm × 1 mm Si_3_N_4_ membrane windows, each covered by a 100 nm-thick Si_3_N_4_ membrane (Norcada, Canada) (Kobayashi *et al.*, 2016*b* ▸).

For both types of specimen disks, both sides of the Si_3_N_4_-membrane windows were coated with a 15–30 nm-thick carbon layer using a JEE-420 vacuum evaporator (Jeol, Japan). To enhance the adhesion of specimen particles to the Si_3_N_4_ membrane, the carbon-coated membrane was treated with a 0.1 mg ml^−1^ solution of poly-l-lysine (PLL) with a molecular weight of approximately 300k (Sigma-Aldrich, USA) (Takayama & Yonekura, 2016 ▸; Kobayashi *et al.*, 2016*b* ▸). After a 30 min treatment, any unbound PLL was rinsed away with distilled water.

### 3.2. Preparation of specimen disks adhering colloidal gold particles

Colloidal gold particles with a mean diameter of 200 nm or 250 nm (BBI Solutions, UK) were dispersed onto the Si_3_N_4_ membranes of each specimen disk. A 30 µL droplet of the colloidal gold particle suspension was applied to the Si_3_N_4_-membrane windows. Once the particles adhered to the membranes, any excess solution was eliminated by blotting. The presence of colloidal gold particles on the Si_3_N_4_ membrane was verified through observation using a TM3000 scanning electron microscope (Hitachi High-Technologies, Japan).

### 3.3. XFEL-XDI experiments

XFEL-XDI experiments were conducted using the diffraction apparatus KOTOBUKI-1 (Nakasako *et al.*, 2013 ▸) or TAKASAGO-6 (Kobayashi *et al.*, 2016*a* ▸) at SACLA. The XFEL pulses had a photon energy of 5.5 keV (corresponding to a wavelength of 0.225 nm) and were provided at a repetition rate of 30 Hz with an approximate duration of 10 fs. The XFEL pulses were focused to an approximate size of 1.5 µm × 1.5 µm (FWHM) using a Kirkpatrick-Baez (K-B) mirror system (Yumoto *et al.*, 2013 ▸), resulting in an intensity of 10^10^–10^11^ photons pulse^−1^ (1.5 µm × 1.5 µm)^−2^. The spatial coherence of each focused X-ray pulse was confirmed to be almost complete using a previously reported method (Kobayashi *et al.*, 2018*b* ▸). Either diffraction apparatus was positioned so that the specimen was within the focal spot of the K-B mirror system. A pair of slit systems, consisting of two pairs of L-shaped silicon frames, was used as guards to eliminate parasitic and background scattering from the upstream X-ray optics.

When using the KOTOBUKI-1 apparatus, the specimen disk was scanned at a speed of 50 µm s^−1^ against the incident XFEL pulses (Nakasako *et al.*, 2013 ▸). In the case of the TAKASAGO-6, the specimen disk mounted on the high-speed translation stage was scanned at a speed of 50 µm/33 ms against the incident XFEL pulses (Kobayashi *et al.*, 2016*a* ▸). Single-shot diffraction patterns were recorded using tandemly placed multi-port CCD (MPCCD) octal and dual detectors (Kameshima *et al.*, 2014 ▸) at distances of 1.6 m and 3.2 m downstream of the specimen position, respectively. The central aperture of the MPCCD octal detector was adjusted to be 8 mm. To reduce intensities in the very small angle region, a 100 µm-thick aluminium-foil attenuator was inserted between a 2.5 mm × 2.5 mm beamstop and the MPCCD dual detector.

### 3.4. Data processing

The diffraction patterns recorded by the two MPCCD detectors were processed using the *SITENNO* program suite (Sekiguchi *et al.*, 2014*a* ▸,*b* ▸) on a high-performance computer (HPC) system at SACLA (Joti *et al.*, 2015 ▸). The suite filtered out noisy and/or low-intensity diffraction patterns that provided limited information about the internal structures of specimen particles. Specifically, for clusters of colloidal gold particles, we selected diffraction patterns with a signal-to-noise ratio higher than 2 at a resolution of 25 µm^−1^ (corresponding to 40 nm in real space).

Prior to analysis, the dark-noise pattern of each detector, recorded just before each scan, was subtracted from the extracted pattern. The beam center position on each detector was determined by quantitatively evaluating the centrosymmetry of the diffraction pattern using *C*_sym_ as defined by Sekiguchi *et al.* (2014*a* ▸),

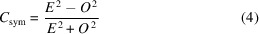

where

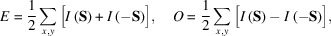


**S** is the scattering vector, *I*(**S**) is the intensity in a targeted region of interest and *I*(−**S**) is that in the symmetry mate with respect to the pixel assumed as the center of the diffraction pattern. Most of the diffraction patterns used in this study displayed *C*_sym_ values greater than 0.8. Subsequently, the diffraction patterns from the two MPCCD detectors were combined to create a single merged diffraction pattern. The maximum resolution of a diffraction pattern was determined as the highest-resolution shell where the signal-to-noise ratios exceeded 2 for more than 200 detector pixels. The resulting diffraction patterns were used in their original form without centrosymmetry averaging.

### 3.5. Ordinary PR calculation

To evaluate the effectiveness of the steering protocol, we conducted PR calculations without implementing the steering protocol (Kodama & Nakasako, 2011 ▸; Oroguchi & Nakasako, 2013 ▸), referred to as ordinary PR calculations, as references. The ordinary PR calculation utilized the hybrid input-output (HIO) algorithm (Fienup, 1982 ▸) and the shrink-wrap (SW) algorithm (Marchesini *et al.*, 2003 ▸). Each PR calculation began with a randomly generated map and a unique initial support, which varied for each individual calculation. The initial support was defined as the region where the absolute values of the autocorrelation function, derived from the diffraction pattern, exceeded 4% of the maximum value.

In the HIO cycle, we used the real-space constraints (Fig. 2 ▸) as

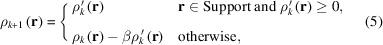

where ρ_*k*_(**r**) and 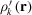 are the maps at the *k*th HIO cycle before and after the reciprocal-space constraint, respectively. Parameter β, to control the update, was set to 0.9. The HIO cycles were iterated 10000 times.

After every 100 HIO cycles, the support was updated by the SW algorithm using a Gaussian low-pass filter, which is defined as

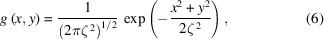

where ζ is the standard deviation and was initially set to 2. The standard deviation used in the *h*th application of the SW (ζ_*h*_) was cycle-dependently reduced as

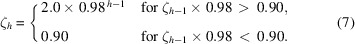

Following the convolution of the PR map with a Gaussian filter, the support was updated by applying a threshold to the filtered map. The threshold was set at 0.04 times the maximum electron density level.

### 3.6. Steered PR calculation

Hereafter, we refer to the PR calculation with the steering protocol as the steered PR calculation. Each steered PR calculation consisted of 10000 HIO cycles, with SW treatments applied every 100 HIO cycles, along with the modification procedure for steering. After the completion of the modification, only HIO cycles were carried out. To accommodate the available workspace limitation for each account, the steered PR calculations using equations (2) and (3) were executed independently and in parallel on ten nodes of 28 cores (280 cores) of the HPC system. We conducted three sets of 280 PR calculations independently.

In the steered PR calculation, the standard deviation of the Gaussian low-pass filter, ζ, was varied depending on the absolute difference between the OS ratios at the ℓth ( 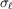 ) and (ℓ−1)th (σ_ℓ−1_) SW operation, which is defined as

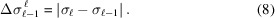

**Figure 3:**
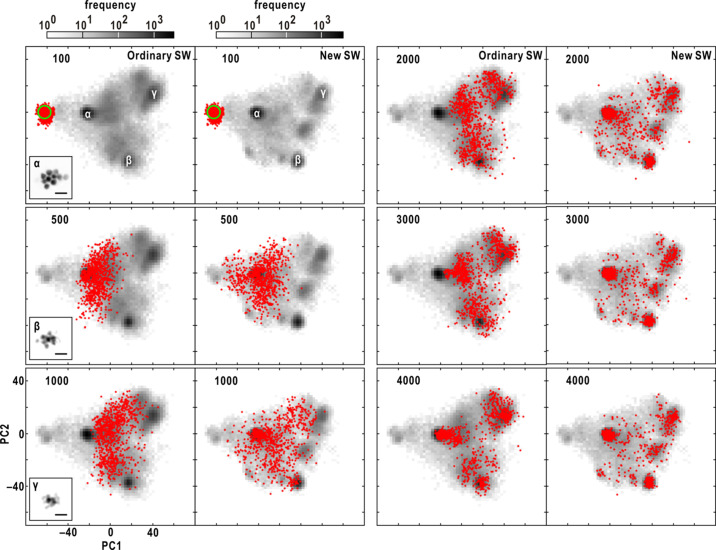
Comparison of the progress of 840 PR calculations using the SW protocol of equation (8), designated new SW (see Section 3.6), with those using the SW protocol of equation (7), designated ordinary SW (details are given in Section 3.5). After an HIO cycle (labeled in the upper left), the red dots indicate the positions of 840 maps on the plane spanned by the PC1 and PC2 vectors from the PCA for the set of 840 final PR maps. The gray-scaled background map in each panel displays how frequently PR maps visited each position on the plane during each set of 840 PR calculations with the scale of the frequency at the top of the panel. The number of HIO cycles is labeled in the upper left of each panel. The green circle in the panel showing the 100th HIO cycle indicates the area of the 840 random maps given for initiating differently and independently in the three sets of 280 PR calculations.

The OS ratio was determined as the ratio of the support area to the total area, which varied depending on the updated size of the support through the SW treatment. In this study, a ζ value of 2 was maintained for 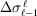 greater than 2, while ζ was set to 0.9 for 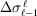 smaller than 2. Once the ζ value reached 0.9, it was maintained for subsequent PR calculations. The threshold for the updated map remained the same as in the ordinary calculation. This new SW update procedure helped to sparsely distribute PR maps in the image space and suppress drastic variations of PR maps in the initial stage of the PR calculation (Fig. 3 ▸).

The first modification for the steered PR calculations was executed after an additional 100 HIO cycles, when ζ of the low-pass filter had reached 0.9 in all 280 parallel calculations. It took between 500 and 1000 HIO cycles for ζ to satisfy this condition in all calculations. At this point, the PR calculation on each core was temporarily paused, and the PR maps were transferred to a core to prepare in ρ_*M*_(*x*, *y*) of equation (2). Each of the 280 PR maps, modified using equation (3), was then returned to its respective core as the initial map for the next HIO cycle.

After the first modification, the modification procedure was iteratively carried out every 500 HIO cycles. Between a pair of the modifications, the support was updated four times using the SW procedure with a ζ value of 0.9. The modification in the steered protocol was finished after the 9000th HIO cycle.

### 3.7. Oversampling smoothness calculations for biological specimens

As described in the *Discussion* [Sec sec5] section, in order to visualize the intricate structures of biological specimens we conducted 250×2 independent PR calculations using the oversampling smoothness (OSS) algorithm (Rodriguez *et al.*, 2013 ▸). The support for the initial map was selected from one of the pairs that exhibited the lowest similarity score in either the ordinary or steered PR calculation.

The real-space constraint in the OSS algorithm was defined as

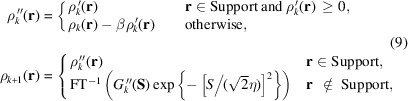

where ρ_*k*_(**r**) is the map at the beginning of the *k*th cycle, and 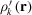 is the inverse Fourier transform (FT^−1^) of the structure factor with the observed amplitude and phase calculated using ρ_*k*_(**r**). ρ_*k*+1_(**r**) is the map generated for the next cycle. β is a weight parameter that was fixed at 0.9 for all the iterations. 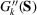 is the Fourier transform of 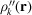 . For maps composed of *m*×*m* pixels, parameter η is linearly decreased from *m* to 1/*m* after every 1000 iterations.

### 3.8. Visualization of trajectory of maps in multidimensional space

When a PR map consists of *J* pixels, it can be represented as a point in a *J*-dimensional space (Sekiguchi *et al.*, 2016 ▸). To visualize the trajectory of the maps in this *J*-dimensional space, we employed PCA on all the final PR maps obtained in the ordinary calculations. Prior to PCA, the maps were optimally superimposed with a correction for the π-rotation. Subsequently, the trajectory of the maps in each PR calculation was projected onto the plane defined by the first and second PC vectors, which predominantly characterized the variation of the PR maps. The maps obtained from the steered calculations were projected onto the plane of the two PC vectors from the ordinary calculations. Additionally, we visualized the frequency at which maps in the PR cycles visited each point on the plane by generating a grayscale map for all the PR calculations.

### 3.9. Computation

All the PR calculations, including the visualization of the map distribution in multidimensional space, were performed on the HPC system at SACLA (Joti *et al.*, 2015 ▸). The computational times for a set of 280 parallelly executed ordinary and steered PR calculations were 48.300 s and 86.226 s, respectively. These times were recorded for a single diffraction pattern that was trimmed to 128 × 128 pixels after 2 × 2 binning. The maximum resolution of the diffraction pattern was 18 µm^−1^ (corresponding to 56 nm in real space).

## 4. Results

To evaluate the effectiveness and efficiency of the steered protocol, we compared the trajectories of PR maps in the image space between the steered and ordinary calculations by focusing on their convergence towards realistic maps. We used diffraction patterns from various aggregates of colloidal gold particles collected using XFEL pulses. The known size and shape of each colloidal particle provided an advantage in assessing the reality of the PR maps.

**Figure 4:**
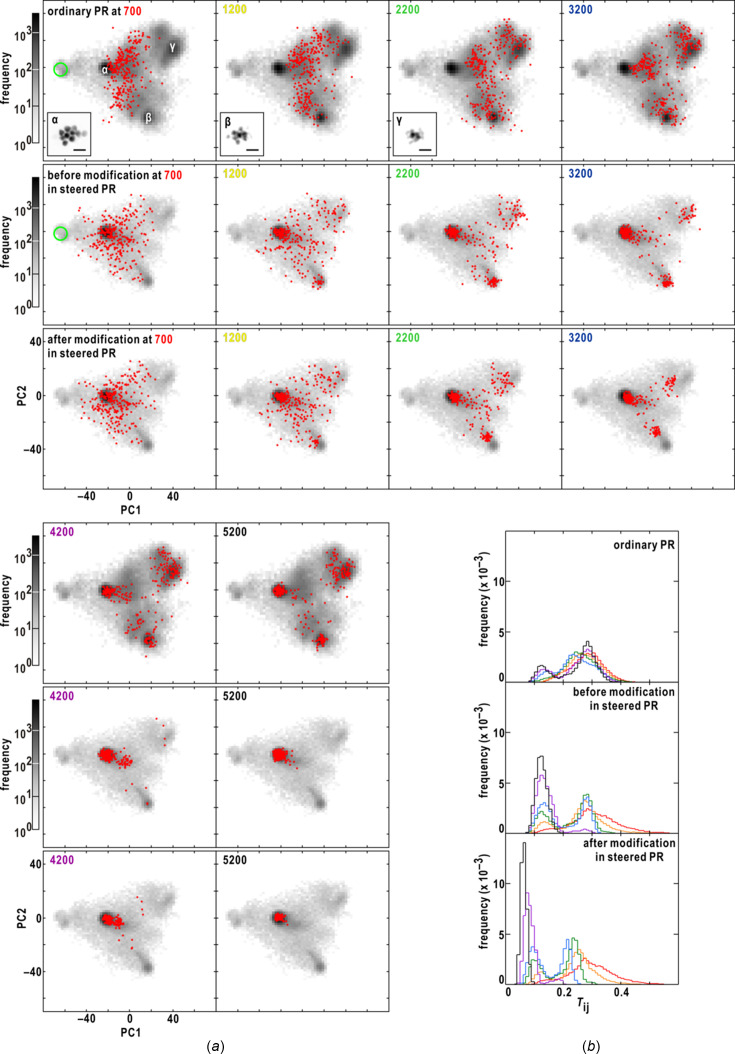
(*a*) The progress of the ordinary and steered PR calculations for the diffraction pattern shown in Fig. 1(*a*). According to the criterion to start the steered protocol (see Section 3.6), the modification using equations (2) and (3) was first applied at the 700th HIO cycle in a set of 280 steered PR calculations. The positions of PR maps at HIO cycles, the colored labels in the upper left corner, are indicated by red dots on the plane spanned by the PC1 and PC2 vectors from the PCA applied to maps at the 10000 HIO cycles in three sets of 280 calculations. The top row shows the results from the ordinary PR calculations. To demonstrate effects of the modification by equations (2) and (3), the distributions of the PR maps before (middle row) and after (bottom row) the modification are shown separately. The gray-scaled map in the background of each panel displays how frequently PR maps visited each position on the plane in the three sets of 280 calculations with the scale of the frequency at the left side of the panel. The green circle in the 700th HIO cycle indicates the area of the 840 random maps given for initiating differently and independently three sets of 280 PR calculations. (*b*) The frequency distributions of the similarity scores among a set of 280 PR maps obtained by each of the ordinary (top panel) and steered (middle and bottom panels) 280 calculations. The colors of the lines correspond to the colors of labels showing the number of HIO cycles in panel (*a*).

Initially, the steered protocol was applied to ten diffraction patterns (Figs. 4 ▸, 5 ▸ and Fig. S1 of the supporting information) that were collected using the KOTOBUKI-1 apparatus. These patterns were previously used to demonstrate the effectiveness of the similarity score for extracting realistic maps in a prior study (Sekiguchi *et al.*, 2017 ▸). Subsequently, the steered protocol was further assessed using 163 diffraction patterns recently collected using the TAKASAGO-6 apparatus, exclusively for this study (Fig. 6 ▸ and Fig. S2 of the supporting information).

We conducted three sets of 280 PR calculations, both with and without the steered protocol. In Figs. 4 ▸–6 ▸ ▸ and Figs. S1 and S2 of the supporting information, the trajectory of maps from one of the three sets is displayed, as the modification process began at different HIO cycles across the three sets. The other two sets yielded similar results to the selected set.

### 4.1. Comparison of the steered and ordinary PR calculations

Fig. 4 ▸(*a*) compares the progress of the steered and ordinary PR calculations for the diffraction pattern shown in Fig. 1 ▸(*a*) on the plane defined by the two PC vectors, which represented the characteristics of all the final projection maps. Realistic maps, where ten colloidal gold particles were clearly distinguishable, were exclusively found in region α, while the maps in the other regions displayed blurred images of colloidal gold particles.

At the 700th cycle, the maps from the steered calculations exhibited a sparser distribution around region α compared with the ordinary calculations. By the 1200th cycle, a significant number of PR maps in the steered calculations remained in region α, whereas the maps in the ordinary PR calculations passed through region α. This disparity between the ordinary and steered calculations can be attributed to the utilization of the new SW algorithm [see equation (8)].

By the 2200th cycle, the maps primarily occupied region α in the steered calculations, while the maps in the ordinary calculations reached regions β and γ. After the 3200th cycle, the maps in the ordinary calculations were distributed within and around all three regions until the completion of the HIO cycle, with approximately 53% of the maps found in region α. In contrast, in the steered calculations, the maps in regions β and γ gradually shifted towards region α, as the maps in region α yielded similarity scores below 0.1 and had a significant influence on equation (2). Notably, the modification using equation (2) was effective in attracting maps from regions β and γ towards region α, as showed by the differences in the map distribution before and after the modifications at the 2200th and 3200th cycles.

Since the protocol relies on the assumption that maps yielding similarity scores smaller than 0.2 may be realistic, we calculated the cycle-dependent variation of frequency distributions based on similarity score values [Fig. 4 ▸(*b*)]. In the ordinary calculations, the distribution displayed multiple peaks, indicating the potential trapping of PR calculations in local minima. In contrast, the distribution in the steered calculations exhibited a single peak after the 4200th cycle, indicating convergence towards a single structure.

**Figure 5:**
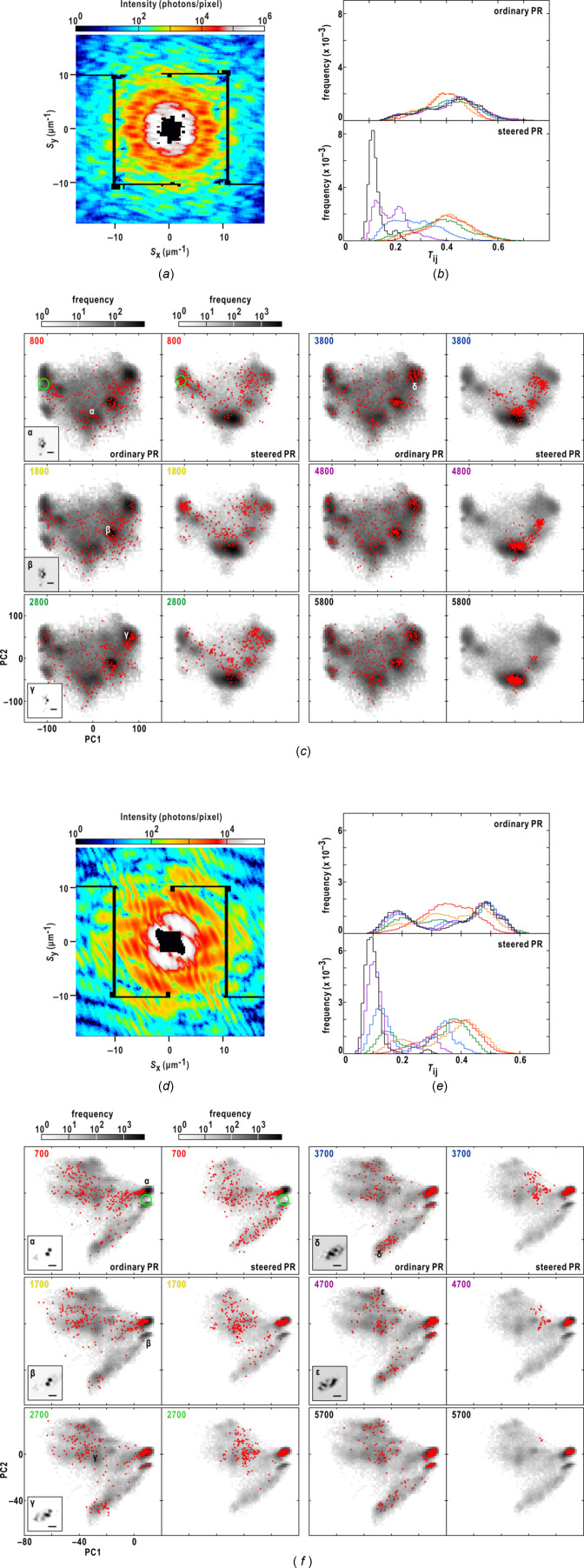
Comparison on the performance of the ordinary and steered calculations for the diffraction patterns from aggregates of colloidal gold particles. (*a*) Diffraction pattern from an aggregate of six colloidal gold particles. The *C*_sym_ value of the pattern was 0.89. The black areas are saturated detector pixels around the beamstop and the gap regions between detector panels. (*b*) The cycle-dependent variations of the frequency distributions of the similarity scores among the maps from a set of 280 ordinary (upper panel) and steered (lower panel) PR calculations. The colors of the lines indicate the HIO cycles in panel (*c*). Regarding the steered calculation, the similarity scores were calculated for maps after the modification at each selected cycle. (*c*) The distribution of the PR maps during the HIO cycles indicated by colored labels in the upper left corner are illustrated as in Fig. 4 ▸(*a*). Projection electron density maps averaged in the labeled regions are shown with a scale bar of 200 nm. (*d*)–(*f*) Results for a diffraction pattern from a specimen composed of two normal and one triangular-shaped colloidal gold particles, the *C*_sym_ of which was 0.84, are illustrated as in panels (*a*)–(*c*). The two diffraction patterns in our previous work (Sekiguchi *et al.*, 2017 ▸) were used with approval from the International Union of Crystallography.

Figs. 5 ▸(*a*)–5(*c*) provide a comparison of the steered and ordinary PR calculations for the diffraction pattern obtained from an aggregate of six colloidal gold particles. In the steered PR calculations, the frequency distributions of similarity scores converged to a single peak centered at approximately 0.1 [Fig. 5 ▸(*b*)], while the distributions in the ordinary calculations remained relatively unchanged. Regarding the distributions of maps on the plane defined by the two PC vectors [Fig. 5 ▸(*c*)], the maps in the ordinary calculations were sparsely distributed until the 1800th cycle, and some maps reached regions β and γ after the 3800th cycle. The maps in region β resembled the realistic map in region α but exhibited slight blurring in the gaps between particles. After the 5800th cycle, the maps became localized in six distinct regions, with the number of maps in region α accounting for less than 27% of the total. In the steered calculations, after the initial sparse distribution until the 2800th cycle, a significant number of maps reached region α, characterized by realistic maps yielding small similarity scores, and attracted other maps towards region α. By the 5800th cycle, almost all maps were concentrated in region α, resulting in a narrow frequency distribution of similarity scores [Fig. 5 ▸(*b*)].

Figs. 5 ▸(*d*)–5(*f*) illustrate the PR calculations performed on a diffraction pattern obtained from two normal and one triangular-shaped colloidal gold particles. In the ordinary calculations, a substantial number of PR maps were located around region α from the 700th to the 1700th cycle [Fig. 5 ▸(*f*)], while some other maps converged towards region β. Both regions exhibited similarities, but region α demonstrated greater reality in terms of the sharpness of the edges of the triangular-shaped particle. The frequency distribution after the 3700th cycle showed two prominent peaks originating from regions α and β, along with minor enhancements [Fig. 5 ▸(*e*)]. In the steered calculation, the maps in region α efficiently attracted the sparsely distributed PR maps towards region α between the 1700th and 4700th cycles [Fig. 5 ▸(*f*)]. By the 5700th cycle, the frequency distribution displayed a single peak centered at *T*_*ij*_ = 0.1 [Fig. 5 ▸(*e*)].

### 4.2. Efficiency to yield realistic maps

**Figure 6:**
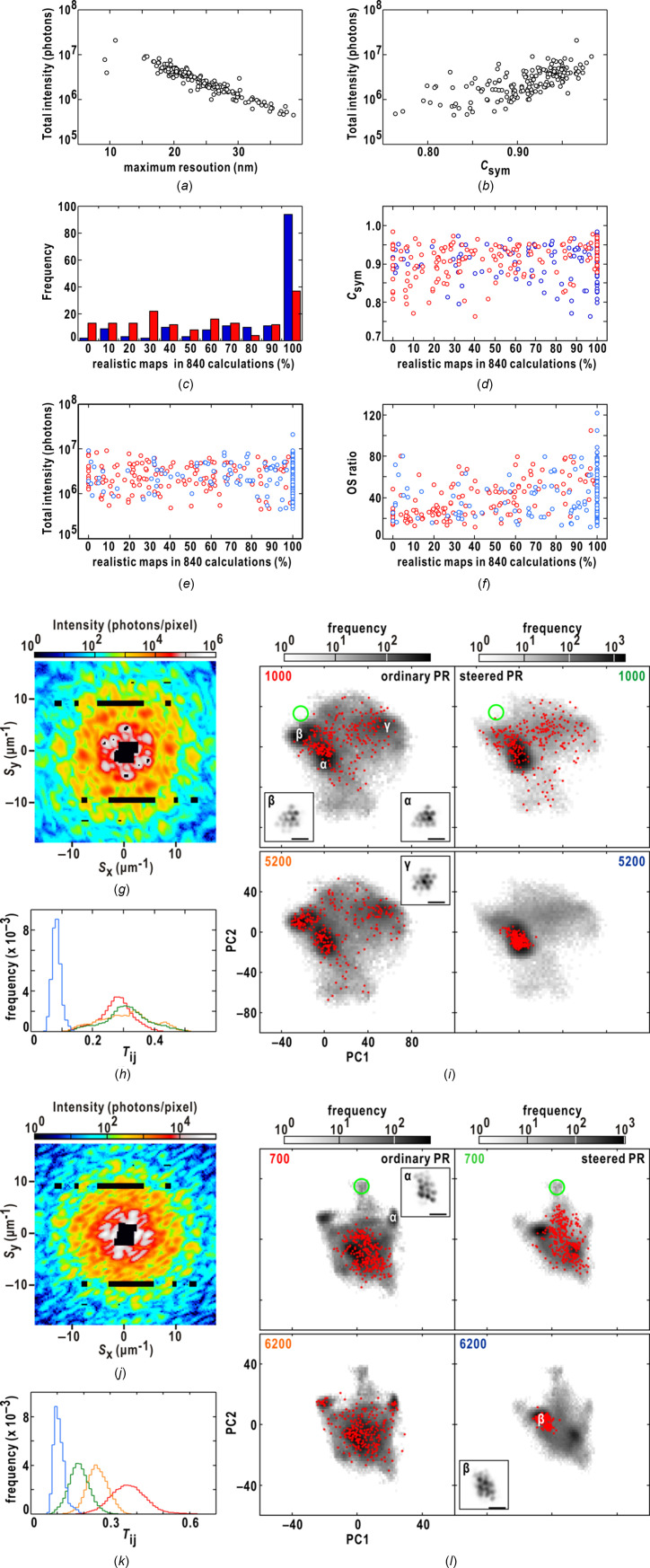
The performance of the steered calculations compared with that of the ordinary calculations for 163 diffraction patterns from various aggregates of colloidal gold particles. For each of the selected 163 diffraction patterns, total intensity is plotted against the maximum resolution (*a*) and *C*_sym_ (*b*). (*c*) Frequencies on the ratio of the number of realistic maps against the three sets of 280 ordinary (red bars) and steered (blue) calculations for 163 diffraction patterns. The number of realistic maps was counted from the results from *K*-means clustering (MacQueen, 1967 ▸) applied to 840 final maps. The *C*_sym_ values (*d*) and the total intensity (*e*) of the diffraction patterns are plotted against the ratio in the ordinary (red open circles) and steered (blue) calculations in panel (*c*). (*f*) The OS ratio of one of the pairs with the smallest similarity score in each set of 840 PR calculations was plotted against the ratio in panel (*c*). Comparison of the steered and ordinary calculations for the diffraction patterns from aggregates of 11 (*g*) and 13 (*j*) colloidal gold particles are illustrated in panels (*h*)–(*i*) and (*k*)–(*l*), respectively, as Fig. 5 ▸. The representative projection maps are shown with scale bars of 600 nm. The *C*_sym_ values of the diffraction patterns in panels (*g*) and (*j*) are 0.93 and 0.89, respectively. In panels (*h*) and (*k*), showing the frequency distributions of the similarity scores, lines are colored as the labels of HIO cycles in panels (*i*) and (*l*), respectively.

To evaluate the efficiency of the steered protocol, we used 163 diffraction patterns obtained from aggregates comprising more than three colloidal gold particles (Fig. 6 ▸ and Fig. S2 of the supporting information). Figs. 6 ▸(*a*)–6(*b*) show the quality of the diffraction patterns. The maximum resolution of each pattern was linearly correlated with the total diffraction intensity. In addition, the centrosymmetry of most diffraction patterns were greater than 0.9, suggesting the good quality of the patterns.

Each diffraction pattern underwent three sets of 280 PR calculations, and the number of realistic maps was determined through manual inspection of the results obtained from *K*-means clustering for 840 retrieved maps on the plane defined by the two PC vectors.

Fig. 6 ▸(*c*) presents the frequency distribution of the ratios between the number of realistic maps and the total number of maps. Although the steered calculations do not always yield realistic maps, the probability of obtaining realistic maps in all 840 steered calculations (100%) was approximately three times greater than that in the ordinary calculations. Furthermore, the steered protocol significantly reduced the probability of generating less than 50% of realistic maps. Interestingly, the probability showed no correlation with the quality of the diffraction patterns in terms of centro-symmetry [Fig. 6 ▸(*d*)] and the total intensity [Fig. 6 ▸(*e*)]. In addition, the probability was almost independent of the OS ratio estimated from the final map displaying the smallest similarity score [Fig. 6 ▸(*f*)].

Figs. 6 ▸(*g*)–6(*i*) provide an example where all three sets of 280 steered calculations resulted in realistic maps. Within a set of 280 steered calculations, the frequency distributions of similarity scores converged to a peak centered around *T*_*ij*_ = 0.09 [Fig. 6 ▸(*h*)]. Each realistic map in region α depicted an aggregate comprising 11 colloidal gold particles [Fig. 6 ▸(*i*)], while maps in regions β and γ displayed ghost densities of particles.

Figs. 6 ▸(*j*)–6(*l*) review a case where few realistic maps were obtained in the steered calculations. The maps in the steered calculations primarily converged to region β, which exhibited blurred images of the maps found in region α in the ordinary calculations [Fig. 6 ▸(*l*)]. However, these maps in region β demonstrated significant similarity to one another, as indicated by the frequency distributions of similarity scores [Fig. 6 ▸(*k*)]. In contrast, the 840 ordinary calculations yielded 180 realistic maps in region α [Fig. 6 ▸(*l*)]. In this case, the maps in the early stages of the steered calculations reaching region β yielded low similarity scores and attracted other maps towards region β.

## 5. Discussion

In this study, we introduced a protocol to steer PR calculations for XDI diffraction patterns, guided by the empirical observation that realistic maps often yield similarity scores below 0.2 (Sekiguchi *et al.*, 2017 ▸). To assess the effectiveness of the protocol, we used diffraction patterns obtained from various aggregates of colloidal gold particles (Figs. 4 ▸–6 ▸ ▸ and Figs. S1 and S2 of the supporting information). Here, we discuss the potential reduction in computational costs achieved by the steered protocol for efficient XDI structure analyses and its applicability to biological specimens with lower electron density contrast compared with colloidal gold particles. Furthermore, we compare the steered protocol with the particle swarm optimization algorithm (PSO) (Kennedy & Eberhart, 1995 ▸; Bonyadi & Michalewicz, 2017 ▸) to explore future possibilities and advancements.

### 5.1. Validation of maps selected using the similarity score

In this study, we applied the steered protocol based on the assumption that the most realistic maps exhibit the smallest similarity score among the retrieved maps. However, there are instances, as depicted in Figs. 6 ▸(*f*)–6(*h*), where pairs of non-realistic maps that differ from the actual particle structures exhibit similarities, leading to a breakdown of this assumption. Therefore, it remains crucial to perform cross-validation using known structural information, such as data from electron and fluorescence microscopy, to validate the maps obtained through the steered protocol. This validation process has been carried out in our previous works (Sekiguchi *et al.*, 2014*a* ▸; Kobayashi *et al.*, 2014 ▸; Takayama *et al.*, 2015*a* ▸; Oroguchi *et al.*, 2018 ▸; Kobayashi *et al.*, 2021 ▸; Uezu *et al.*, 2023 ▸).

### 5.2. Reduction of computational cost for PR calculations

In the case of the ordinary protocol, we typically carried out 1000 independent PR calculations with 10000 HIO cycles for a single diffraction pattern. Consequently, there is a need to reduce the number of PR calculations to efficiently extract realistic maps, especially in XFEL-XDI experiments where numerous diffraction patterns are collected within a short timeframe (Kobayashi *et al.*, 2016*a* ▸; Nakasako, 2018 ▸).

The performance of the steered protocol in the *Results* section[Sec sec4] (Figs. 4 ▸–6 ▸ ▸ and Figs. S1 and S2 of the supporting information) demonstrates the potential of the steered protocol to decrease both the number of trial calculations and the required number of HIO cycles for obtaining realistic maps. In cases where realistic maps were obtained, more than 80% of the 100 steered calculations reached the region of realistic maps within the 4000–5500th HIO cycle. The computational time for executing 5000 HIO cycles on each core in the steered calculations is 43 s (see Section 3.9). In XFEL-XDI experiments utilizing the TAKASAGO-6 diffraction apparatus (Fig. 6 ▸), the proportion of complex diffraction patterns from aggregates of colloidal gold particles in the irradiation area was less than 0.1% of the used XFEL pulses. Considering that approximately 31000 XFEL pulses can be utilized within an hour at SACLA (Nakasako, 2018 ▸), the computational time and automatic suggestion of realistic maps using the steered PR calculations could enable semi-online structure analysis during XFEL-XDI experiments.

The number of HIO cycles required for the convergence of steered PR calculations towards the region of realistic maps depends on various factors, including the sizes of the specimen particles, incident X-ray intensity, signal-to-noise ratio, area of detector saturation, and OS ratio of each diffraction pattern. Once the steered calculations reach convergence, the frequency distribution of similarity scores typically exhibits a single peak centered within the range 0.05 < *T*_*ij*_ < 0.2 and with a narrow full width at half-maximum of less than 0.1 [Figs. 4 ▸(*b*), 5 ▸(*b*), 5 ▸(*e*), 6 ▸(*h*) and 7 ▸(*c*)]. Thus, the frequency distribution of similarity scores can serve as a measure for determining the termination timing. Implementing an automatic termination protocol for sets of parallelly executed steered calculations has the potential to further reduce computational time.

### 5.3. Application to biological specimens

The steered PR calculation proved advantageous in obtaining realistic maps from diffraction patterns of colloidal gold particle aggregates, likely due to the electron density contrast of the particles against the Si_3_N_4_ membrane (or their large scattering cross-section). In our XFEL-XDI experiments, we have focused on investigating the structures of frozen-hydrated biological cells and cellular organelles in vitreous ice (Takayama *et al.*, 2015*a* ▸; Oroguchi *et al.*, 2015 ▸; Nakasako *et al.*, 2020 ▸; Kobayashi *et al.*, 2021 ▸; Uezu *et al.*, 2023 ▸). However, the low electron density contrast of biological specimens against vitreous ice and the Si_3_N_4_ membrane results in weak diffraction intensities compared with colloidal gold particles.

**Figure 7:**
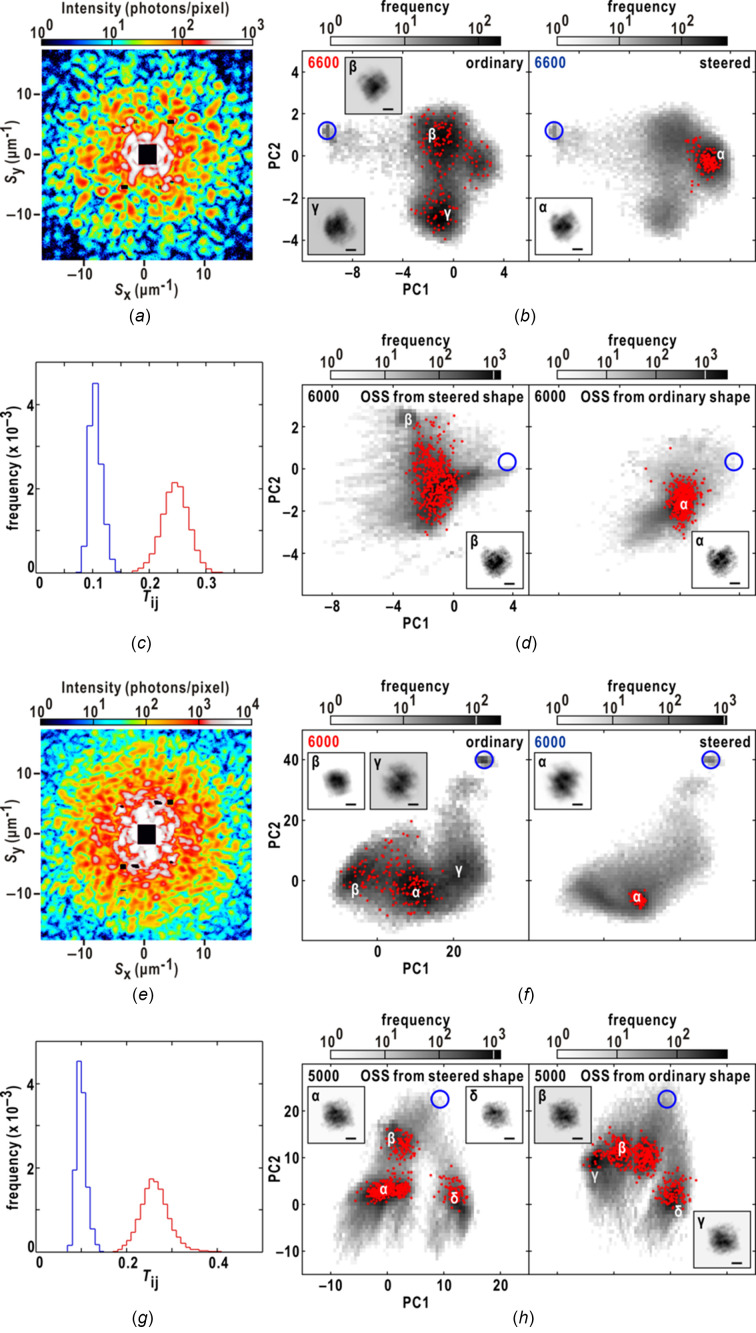
Comparison of the performance of the ordinary and steered PR calculations for the diffraction patterns [(*a*) and (*e*)] from the nuclei of budding yeast. (*b*) The distribution of 160 maps at the 6600th HIO cycle in the ordinary (left panel) and steered (right panel) PR calculations as illustrated in Fig. 5 ▸. Representative electron density maps are depicted with scale bars of 300 nm. (*c*) The frequency distributions of the similarity scores among the maps from a set of 160 ordinary (red line) and steered (blue line) PR calculations at the 6600th HIO cycle. (*d*) The distribution of the maps at the 6000th OSS cycle illustrated in the same manner as panel (*b*). The left panel shows the distributions of the OSS maps, which started from the support of one of the maps displaying the smallest similarity score among the three sets of 160 ordinary PR calculations. The right panel shows the distribution of OSS maps, which started from the support of one of the maps displaying the smallest similarity scored in the three sets of 160 steered calculations. Representative electron density maps are shown with scale bars of 300 nm. Panels (*f*)–(*h*) show the results for the diffraction pattern in (*e*) as panels (*a*)–(*d*).

In this context, we examined the feasibility of the steered protocol in PR calculations for diffraction patterns obtained from the nuclei of budding yeast (*Saccharomyces cerevisiae*) in the interphase of the cell cycle, serving as a representative biological specimen (Fig. 7 ▸) (Uezu *et al.*, 2023 ▸). The interphase nuclei were approximated as ellipsoids with a short axis of 340 nm and a long axis of 420 nm. In a previous study, ordinary calculations yielded realistic maps for 373 out of 1333 diffraction patterns recorded beyond a resolution of 25 µm^−1^ (corresponding to 40 nm in real space) with signal-to-noise ratios higher than 3.

Figs. 7 ▸(*a*)–7(*d*) compare the results obtained from the steered and ordinary calculations for a diffraction pattern from a nucleus. The steered calculations converged to region α, while the maps in the ordinary calculations were dispersed across three regions. In both the final maps of the ordinary and steered calculations, we selected one of the pairs with the smallest similarity score as the initial support for the subsequent PR calculations using the OSS (Rodriguez *et al.*, 2013 ▸). The OSS maps, starting from different supports [Fig. 7 ▸(*d*)], exhibited similar fine structures within the nucleus, which were similar to each other and consistent with our proposed model for the distribution of chromosomes within the nuclei (Uezu *et al.*, 2023 ▸).

Figs. 7 ▸(*e*)–7(*h*) present another example in which the convergence of maps differed among the three sets of 160 steered calculations. The ordinary calculations yielded maps with greater diversity compared with the steered calculations. When validating the maps based on our putative model of the nuclear structure (Uezu *et al.*, 2023 ▸), the maps in region α were found to be more accurate than the others. The OSS calculations, utilizing the support from one of the maps with the smallest similarity score, produced an image of the nucleus consistent with the putative model [Fig. 7 ▸(*h*)].

The implementation of the steered protocol on biological specimens revealed that it facilitated faster convergence to realistic maps. However, the probability of achieving convergence to realistic maps was comparatively lower for specimens with low electron density contrast [Figs. 7 ▸(*e*)–7(*h*)]. This indicates that there is room for improvement in equations (2) and (3) for electron density modification, as well as equation (8) for SW, to enhance the convergence to realistic maps for biological specimens.

### 5.4. Relation between steered protocol and swarm optimization algorithm

The PSO algorithm (Kennedy & Eberhart, 1995 ▸; Bonyadi & Michalewicz, 2017 ▸) is used to iteratively search for the optimal position of particles representing variables in a target system. The algorithm facilitates communication among the particles by utilizing their positions and velocities from previous iterations. The similarity-score weighted update of maps in the steered protocol may resemble the communication in the PSO algorithm, but ignores the velocities of the PR maps in the multidimensional space.

Figs. 6 ▸(*j*)–6(*l*) and 7(*e*)–7(*h*) may provide clues for the improvement in the current version of the steering protocol when applied to weak diffraction patterns. To increase the efficiency to yield realistic maps, we can draw an analogy with the PSO algorithm and introduce velocity terms for maps between iteration cycles in equations (1) and (2). Additionally, it is essential to evaluate whether a cycle-dependent weight *w* in equation (2) outperforms random variation, as seen in the PSO algorithm. By making these adjustments to the steered protocol, we can enhance the likelihood of generating realistic maps.

## Supplementary Material

Diffraction pattern and phase-retrieval calculations supporting the conclusion in the main text. DOI: 10.1107/S1600577523009864/yn5104sup1.pdf
