# Supplementary material for: Protocol using similarity score and improved shrink-wrap algorithm for better convergence of phase-retrieval calculation in X-ray diffraction imaging
Source: J Synchrotron Radiat. 2024 Jan 1;31(Pt 1):113–28. doi: 10.1107/S1600577523009864 (PMC10833425; doi:10.1107/S1600577523009864)
Supplement: Supplementary file 1 [file s-31-00113-sup1.pdf]

JOURNAL OF SYNCHROTRON RADIATION

Volume 31 (2024)

Supporting information for article:

Protocol using similarity score and improved shrink-wrap algorithm for better convergence of phase-retrieval calculation in X-ray diffraction imaging

Syouyo Yoshida, Kosei Harada, So Uezu, Yuki Takayama and Masayoshi Nakasako

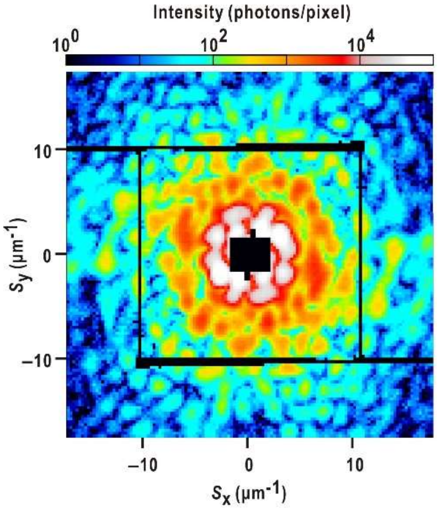
Intensity (photons/pixel)
10^0 10^2 10^4
S_y (μm^{-1})
S_x (μm^{-1})

(a)

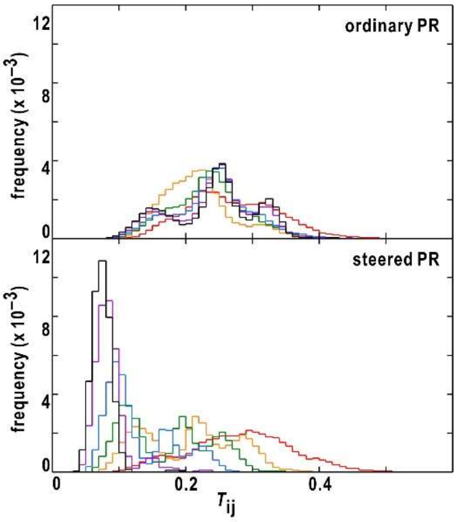
ordinary PR
steered PR
frequency (x 10^{-3})
T_{ij}

(b)

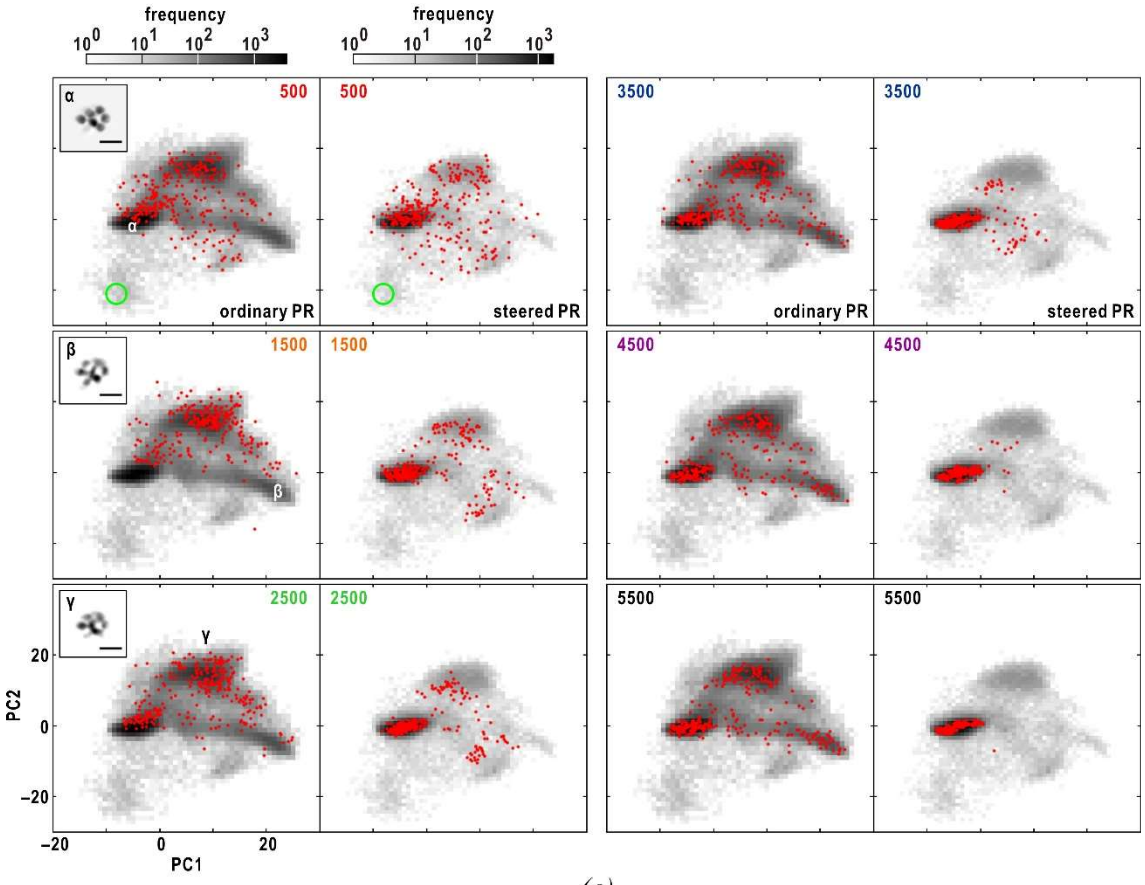
frequency
10^0 10^1 10^2 10^3
500
1500
2500
3500
4500
5500
ordinary PR
steered PR
α
β
γ
PC1
PC2

(c)

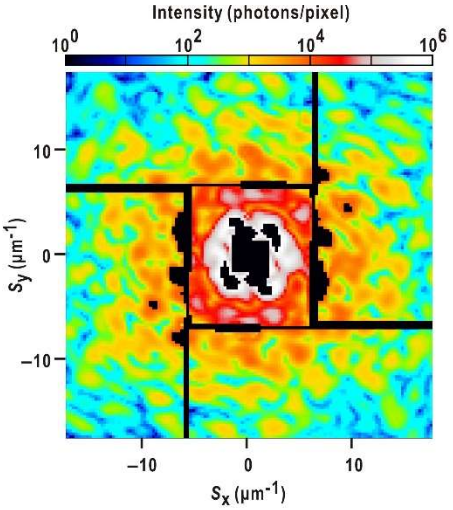
Intensity (photons/pixel)
10^0 10^2 10^4 10^6
s_y (μm^{-1})
s_x (μm^{-1})
10
0
−10
−10
0
10

(d)

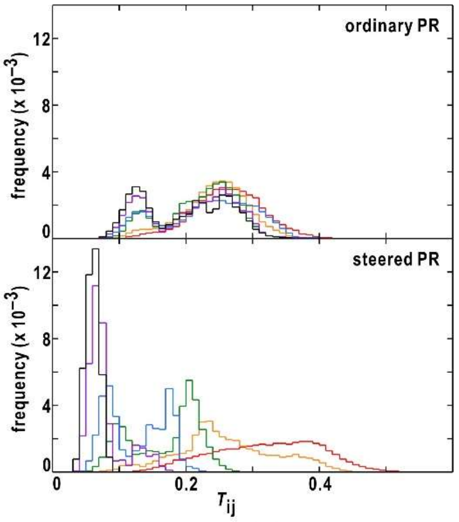
ordinary PR
steered PR
frequency (x 10^{-3})
12
8
4
0
0
0.2
0.4
T_{ij}

(e)

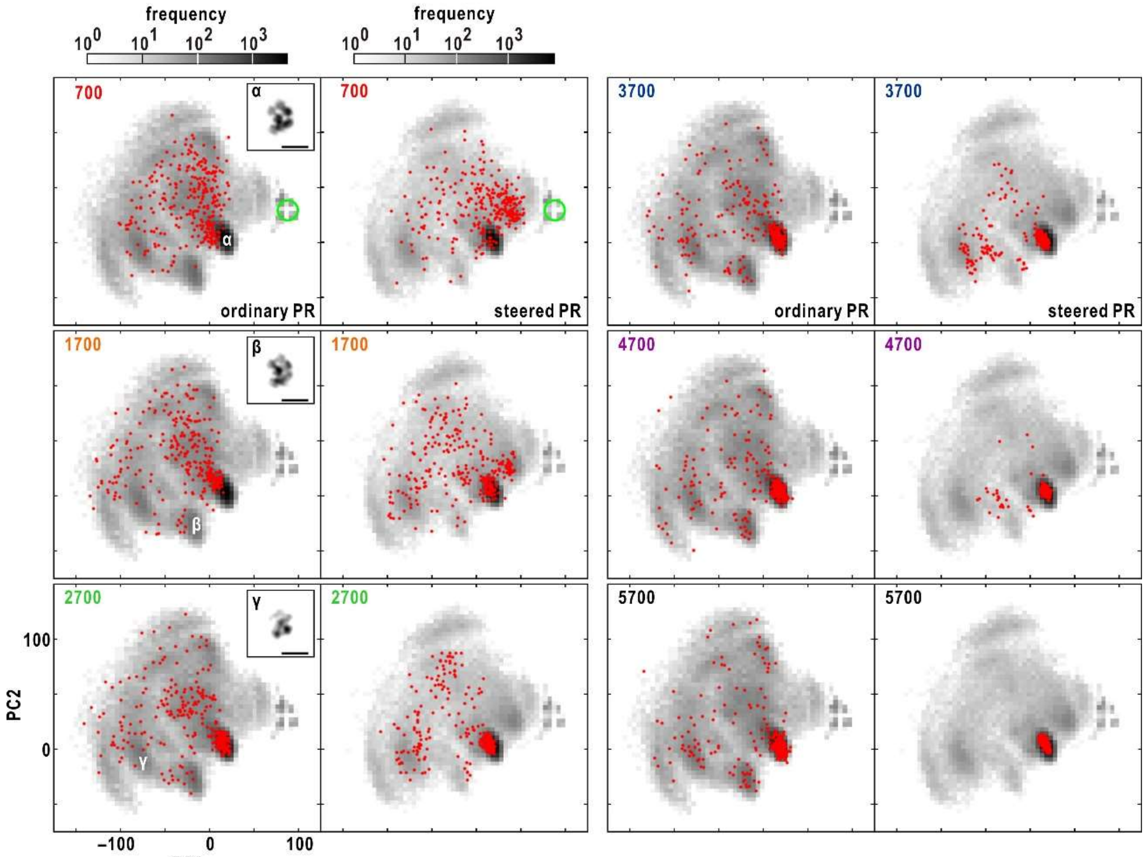
frequency
10^0 10^1 10^2 10^3
700
1700
2700
3700
4700
5700
ordinary PR
steered PR
α
β
γ
PC2
PC1
100
0
−100
0
100

(f)

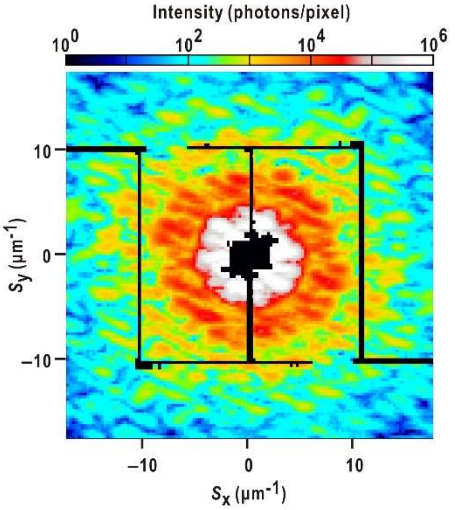
Intensity (photons/pixel)
10^0 10^2 10^4 10^6
s_y (μm^{-1})
s_x (μm^{-1})
10
0
−10

(g)

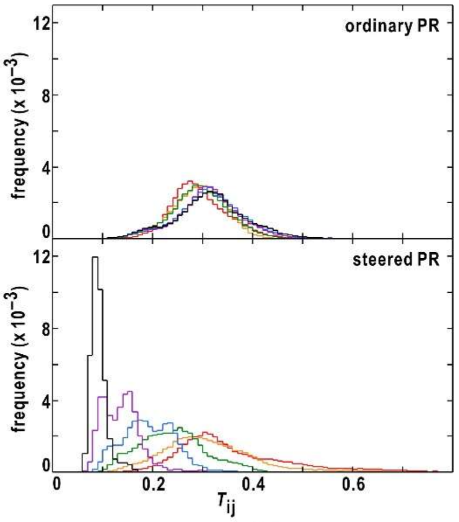
ordinary PR
steered PR
frequency (x 10^{-3})
T_{ij}
0
0.2
0.4
0.6
12
8
4

(h)

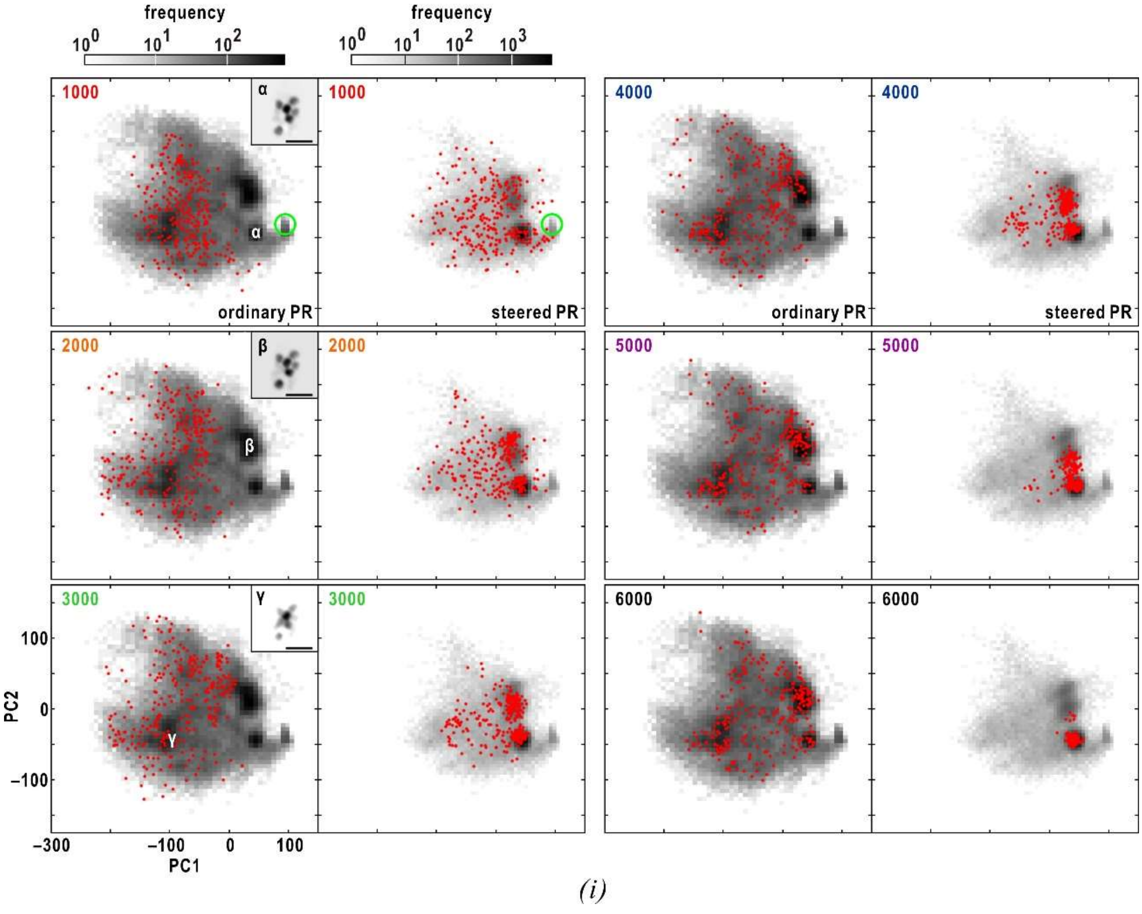
frequency
10^0 10^1 10^2
10^0 10^1 10^2 10^3
1000
2000
3000
4000
5000
6000
α
β
γ
ordinary PR
steered PR
PC1
PC2
−300
−100
0
100
−100

(i)

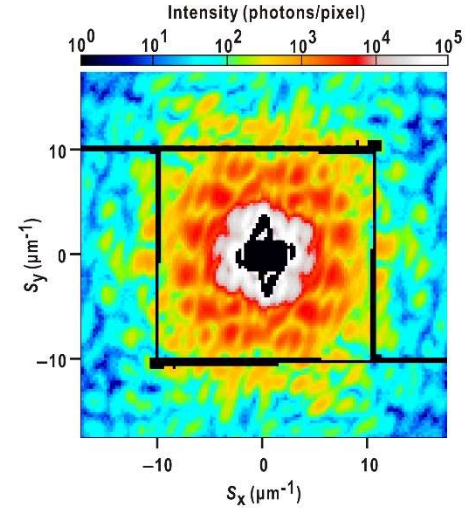
Intensity (photons/pixel)
10^0 10^1 10^2 10^3 10^4 10^5
S_y (μm^{-1})
S_x (μm^{-1})
10
0
−10

(j)

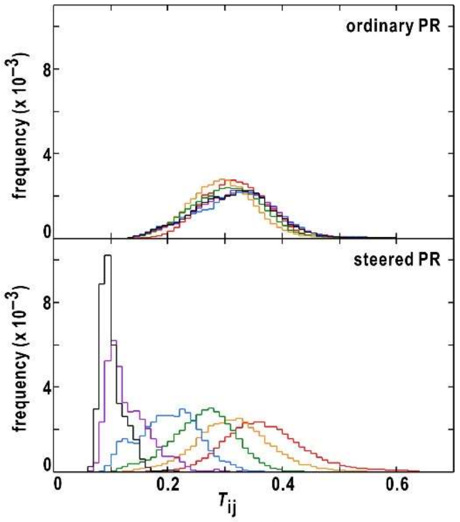
ordinary PR
steered PR
frequency (x 10^{-3})
8
4
0
0.2
0.4
0.6
T_{ij}

(k)

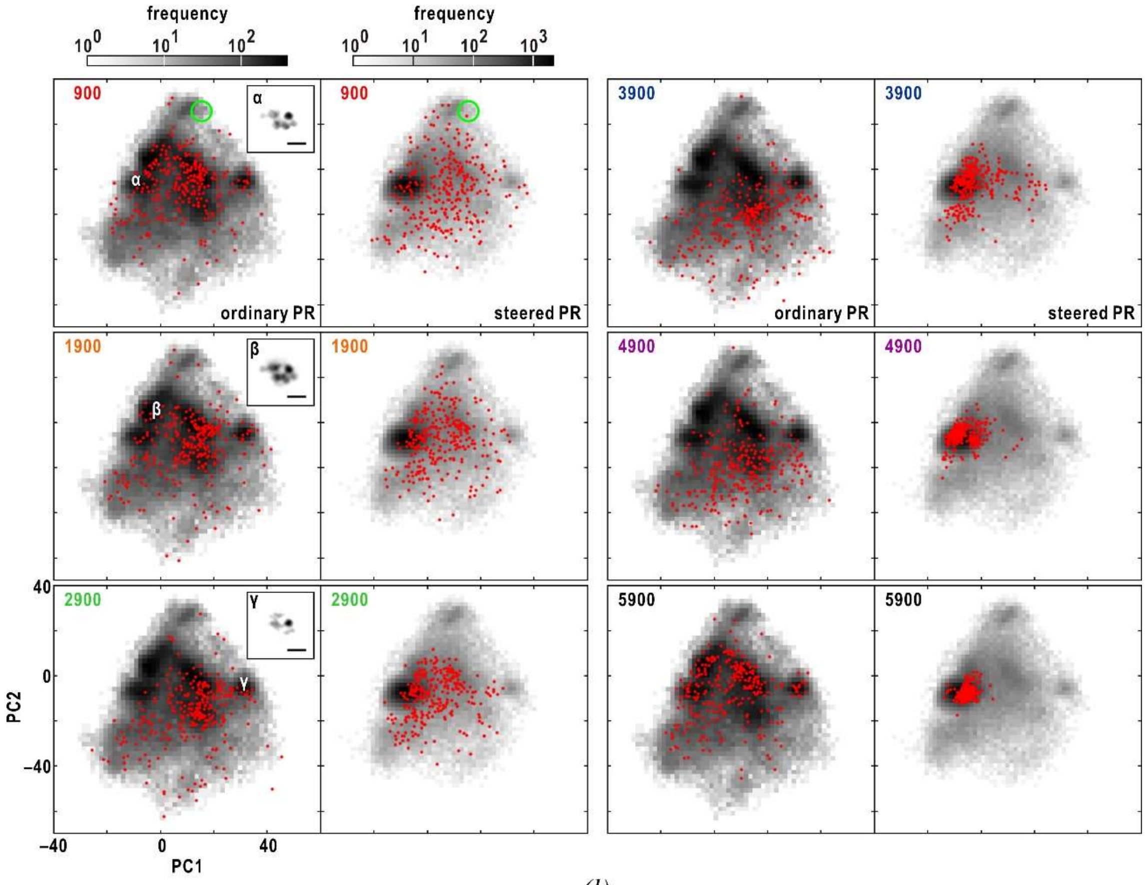
frequency
10^0 10^1 10^2
10^0 10^1 10^2 10^3
900
3900
1900
4900
2900
5900
α
β
γ
ordinary PR
steered PR
PC2
PC1
40
0
−40

(l)

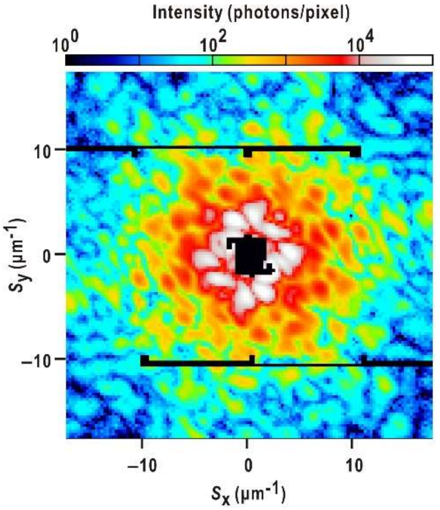
Intensity (photons/pixel)
10^0
10^2
10^4
S_y (μm^{-1})
S_x (μm^{-1})

(m)

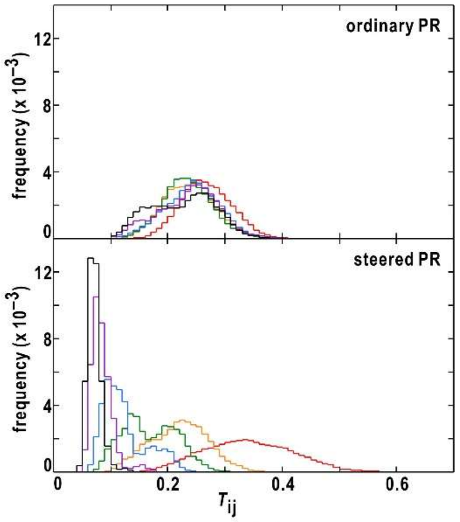
ordinary PR
steered PR
frequency (x 10^{-3})
T_{ij}

(n)

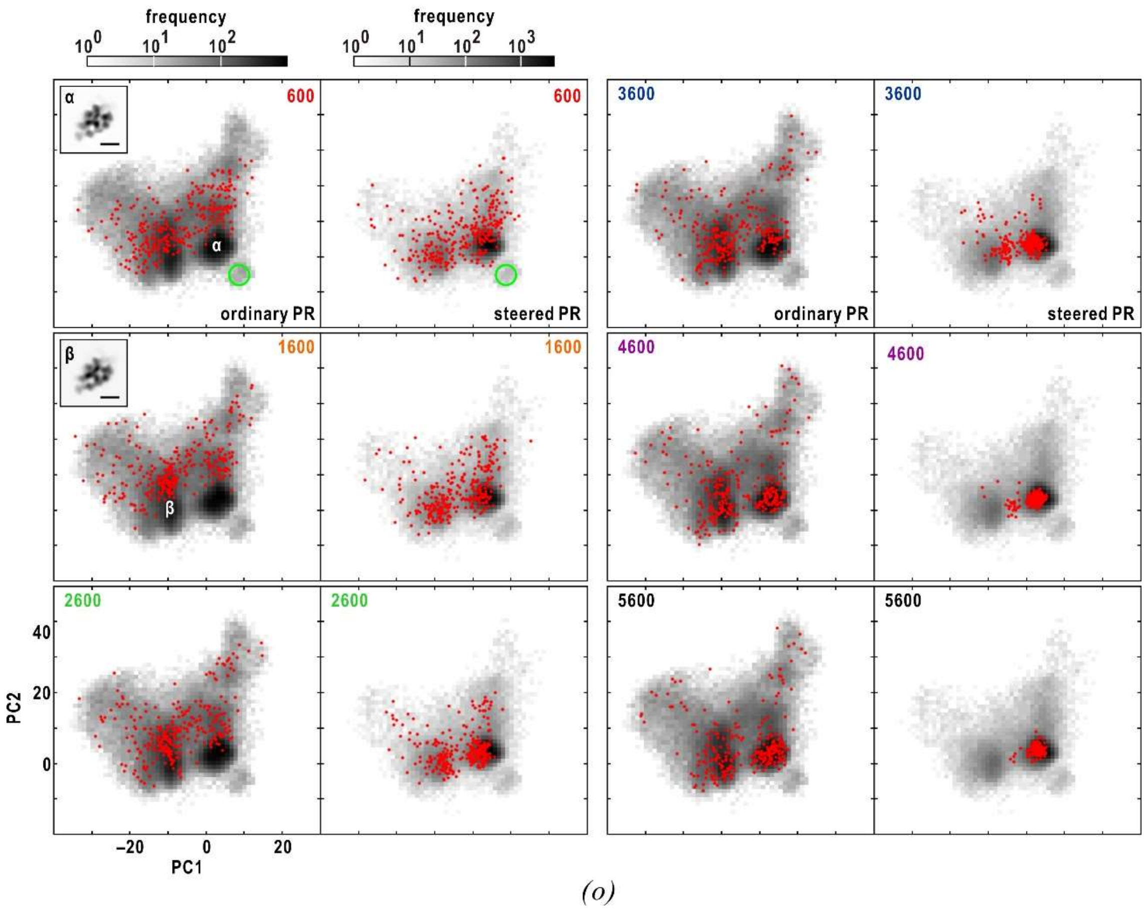
frequency
frequency
600
1600
2600
3600
4600
5600
ordinary PR
steered PR
α
β
PC1
PC2

(o)

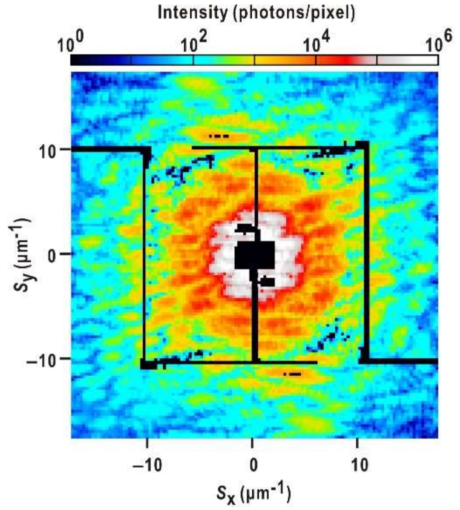
Intensity (photons/pixel)
10^0
10^2
10^4
10^6
S_y (μm^{-1})
S_x (μm^{-1})
10
0
−10
−10
0
10

(p)

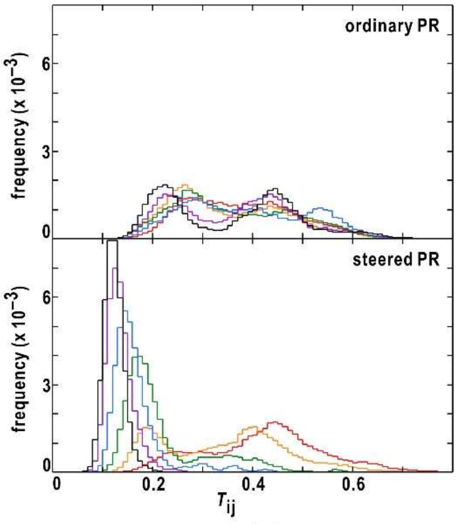
ordinary PR
steered PR
frequency (x 10^{-3})
frequency (x 10^{-3})
6
3
0
6
3
0
0
0.2
0.4
0.6
T_{ij}

(q)

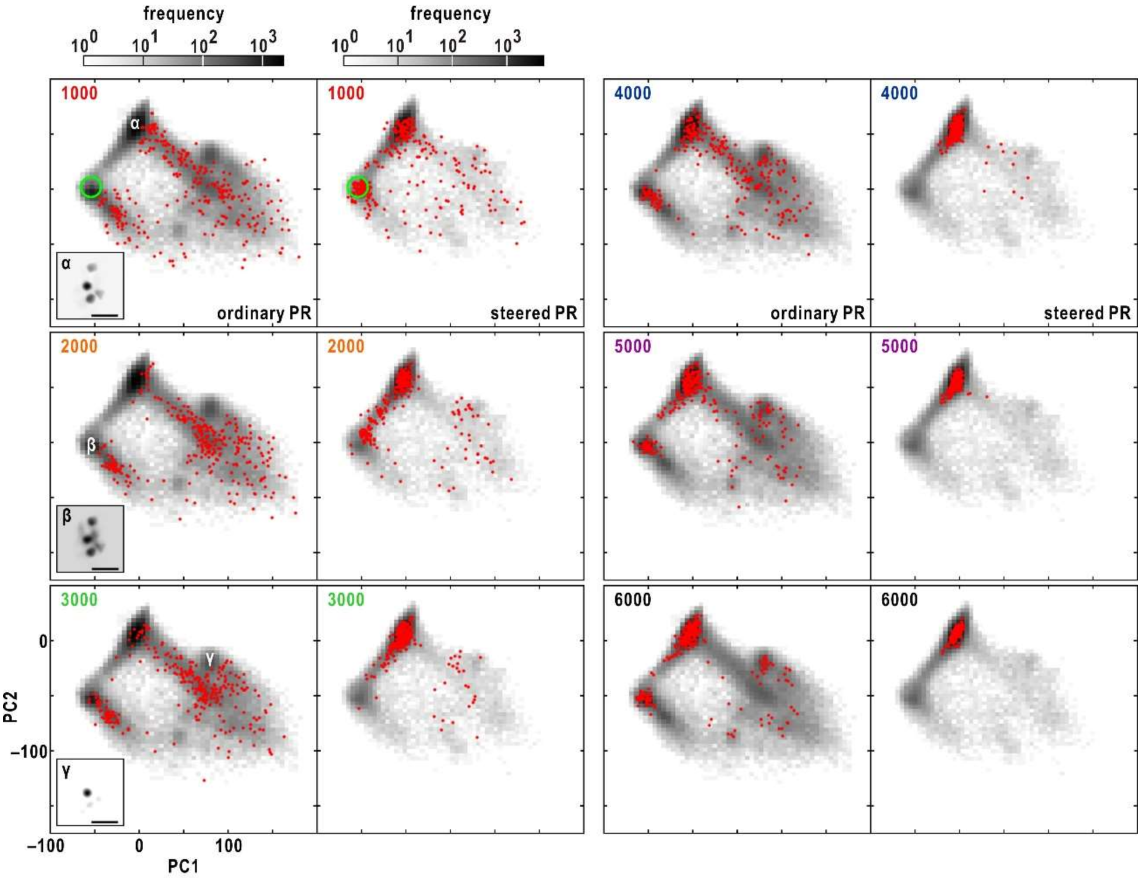
frequency
10^0
10^1
10^2
10^3
frequency
10^0
10^1
10^2
10^3
1000
α
1000
4000
4000
ordinary PR
steered PR
ordinary PR
steered PR
2000
β
2000
5000
5000
3000
γ
3000
6000
6000
PC2
0
−100
−100
0
100
PC1

(r)

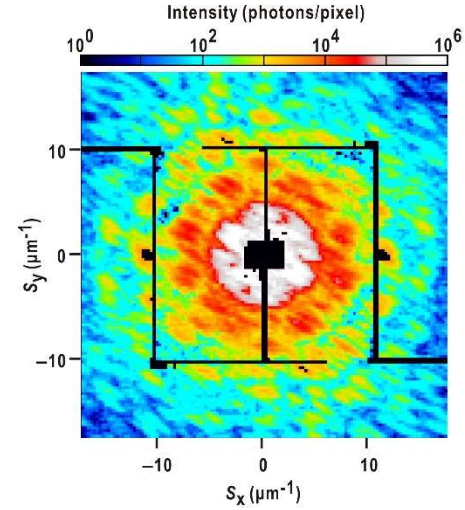

(s)

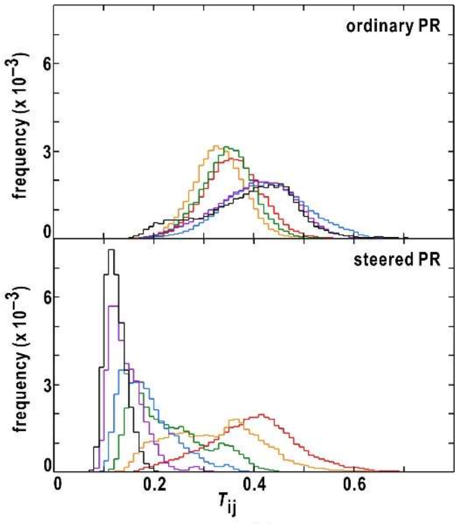

(t)

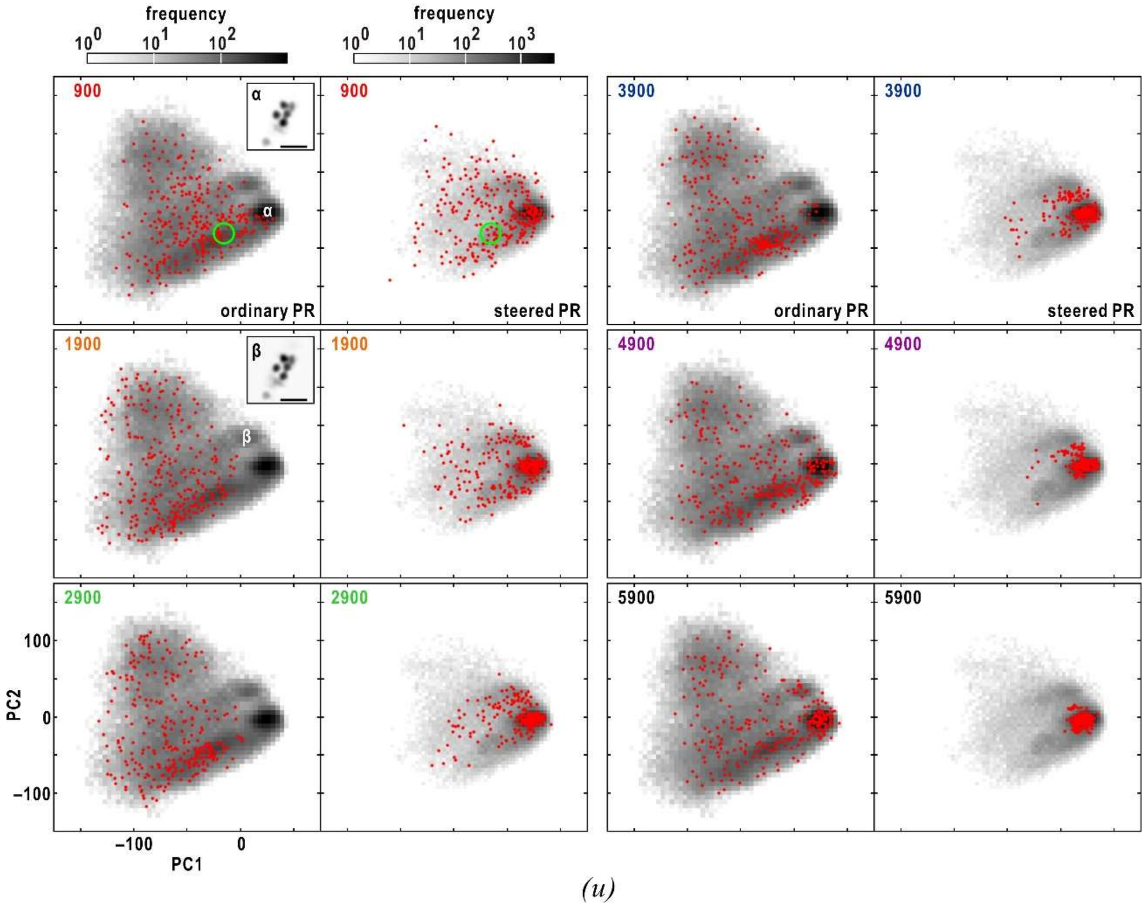

(u)

Figure S1

Comparison of the performance of the ordinary and steered calculations for the seven diffraction patterns from aggregates of colloidal gold particles (panels (a), (d), (g), (j),

(m), (p), and (s)), with the C_{sym} values greater than 0.85. The progress of the PR calculations regarding the similarity scores (panels (b), (e), (h), (k), (n), (q) and (t)) and the distributions of maps in the representative two-dimensional planes (panels (c), (f), (i), (l), (o), (r) and (u)) are illustrated as in Figure 5 in the main text. Projection electron density maps averaged in the labeled regions (panels (c), (f), (i), (l), (o), (r) and (u)) are depicted with a scale bar of 200 nm. The seven diffraction patterns in our previous work (Sekiguchi *et al.*, 2017; doi: 10.1107/S1600577517008396) were reused with approval from Journal of Synchrotron Radiation and The International Union of Crystallography.

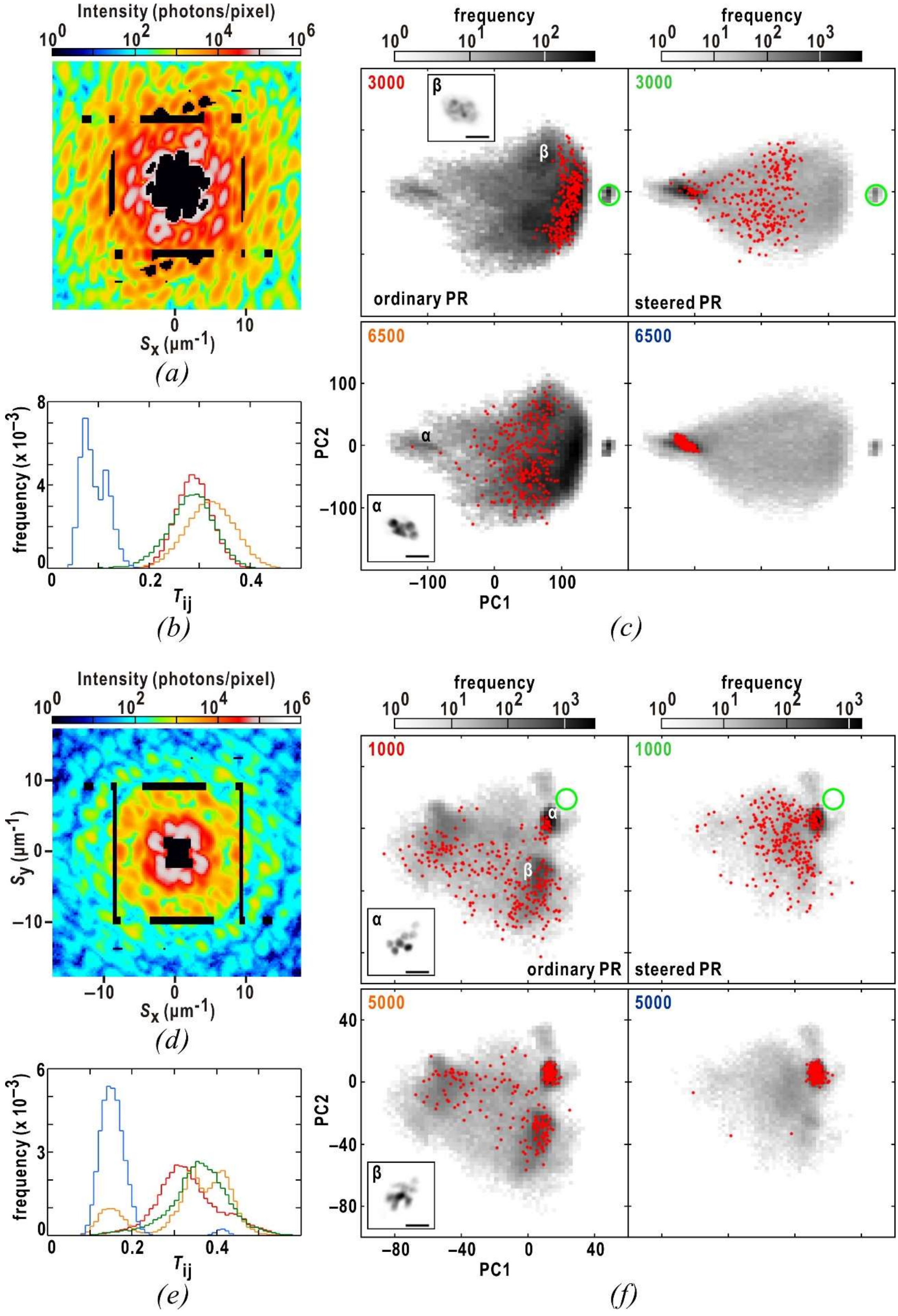
Intensity (photons/pixel)
10^0 10^2 10^4 10^6
0 10
S_x (μm^{-1})
(a)
frequency (x 10^{-3})
8 4 0
0 0.2 0.4
T_{ij}
(b)
frequency
10^0 10^1 10^2
frequency
10^0 10^1 10^2 10^3
3000
β
ordinary PR
3000
steered PR
6500
α
6500
PC2
100 0 −100
−100 0 100
PC1
(c)
Intensity (photons/pixel)
10^0 10^2 10^4 10^6
S_y (μm^{-1})
10 0 −10
−10 0 10
S_x (μm^{-1})
(d)
frequency (x 10^{-3})
6 3 0
0 0.2 0.4
T_{ij}
(e)
frequency
10^0 10^1 10^2 10^3
frequency
10^0 10^1 10^2 10^3
1000
α
β
ordinary PR
1000
steered PR
5000
5000
PC2
40 0 −40 −80
−80 −40 0 40
PC1
(f)

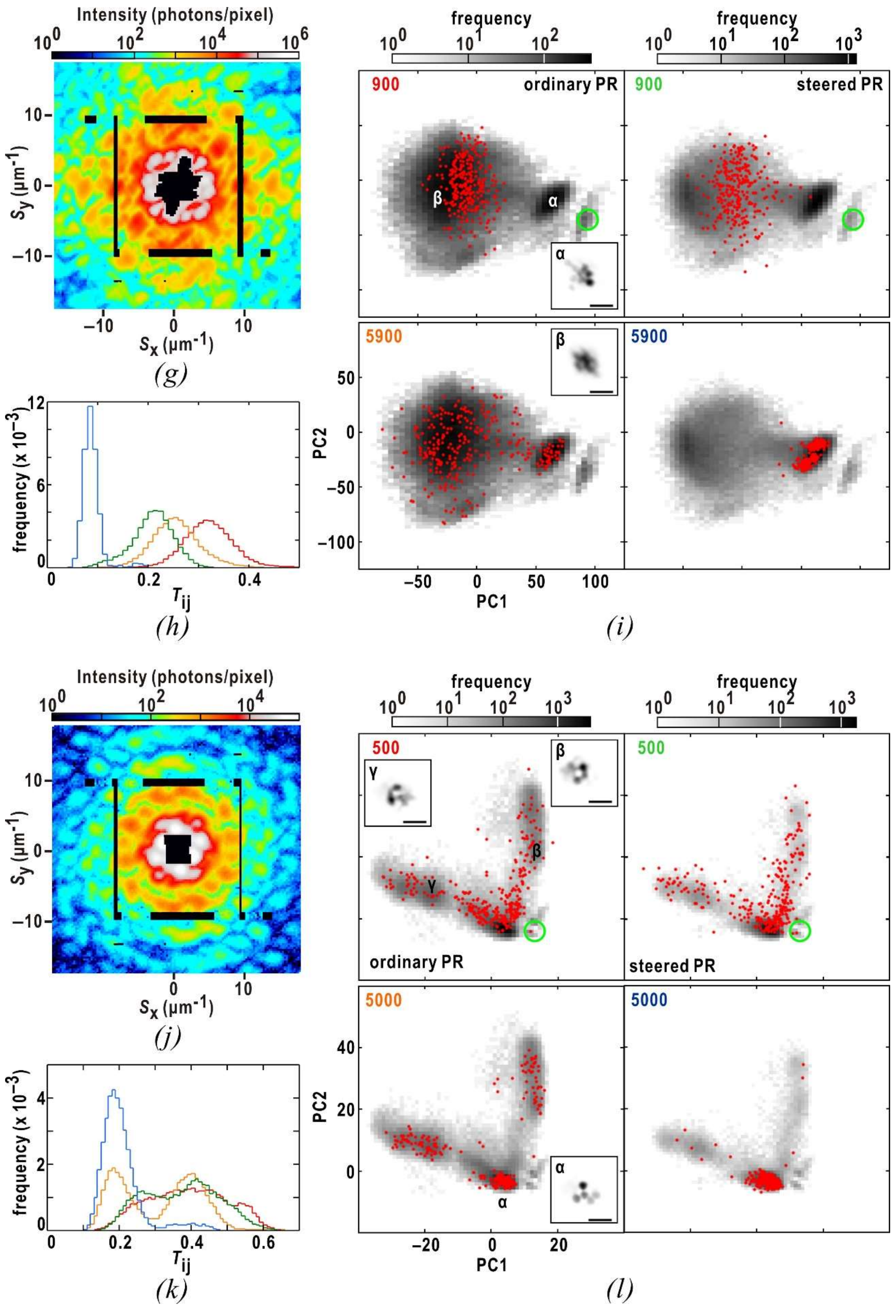
Intensity (photons/pixel)
frequency
900
ordinary PR
steered PR
5900
PC1
PC2
S_x (μm^{-1})
S_y (μm^{-1})
frequency (x 10^{-3})
T_{ij}
(g)
(h)
(i)
500
5000
(j)
(k)
(l)

Figure S2

Comparison of the steered and ordinary PR calculations for selected four out of 163 diffraction patterns from aggregates of colloidal gold particles are illustrated as Figures 7(c-e) and (f-h) in the main text.
